# New Hybrid Compounds Combining Fragments of Usnic Acid and Thioether Are Inhibitors of Human Enzymes TDP1, TDP2 and PARP1

**DOI:** 10.3390/ijms222111336

**Published:** 2021-10-20

**Authors:** Nadezhda S. Dyrkheeva, Aleksandr S. Filimonov, Olga A. Luzina, Kristina A. Orlova, Irina A. Chernyshova, Tatyana E. Kornienko, Anastasia A. Malakhova, Sergey P. Medvedev, Alexandra L. Zakharenko, Ekaterina S. Ilina, Rashid O. Anarbaev, Konstantin N. Naumenko, Kristina V. Klabenkova, Ekaterina A. Burakova, Dmitry A. Stetsenko, Suren M. Zakian, Nariman F. Salakhutdinov, Olga I. Lavrik

**Affiliations:** 1 Institute of Chemical Biology and Fundamental Medicine, Siberian Branch of the Russian Academy of Sciences, 630090 Novosibirsk, Russia; dyrkheeva.n.s@gmail.com (N.S.D.); chernyshova0305@gmail.com (I.A.C.); t.kornienko1995@gmail.com (T.E.K.); amal@bionet.nsc.ru (A.A.M.); medvedev@bionet.nsc.ru (S.P.M.); a.zakharenko73@gmail.com (A.L.Z.); katya.plekhanova@gmail.com (E.S.I.); anarbaev@niboch.nsc.ru (R.O.A.); k-naumenko@mail.ru (K.N.N.); zakian@bionet.nsc.ru (S.M.Z.); 2 N.N. Vorozhtsov Novosibirsk Institute of Organic Chemistry, Siberian Branch of the Russian Academy of Sciences, 630090 Novosibirsk, Russia; alfil@nioch.nsc.ru (A.S.F.); luzina@nioch.nsc.ru (O.A.L.); anvar@nioch.nsc.ru (N.F.S.); 3 Department of Natural Sciences, Novosibirsk State University, 630090 Novosibirsk, Russia; kristina-orlova1999@mail.ru (K.A.O.); k.klabenkova@g.nsu.ru (K.V.K.); e.burakova1@nsu.ru (E.A.B.); d.stetsenko@nsu.ru (D.A.S.); 4 Federal Research Centre Institute of Cytology and Genetics, Siberian Branch of the Russian Academy of Sciences, 630090 Novosibirsk, Russia; 5 Department of Physical and Chemical Biology and Biotechnology, Altai State University, 656049 Barnaul, Russia

**Keywords:** usnic acid, thioether, tyrosyl-DNA phosphodiesterase 1, TDP1 inhibitor, inhibiting activity, TDP2, PARP1, topotecan, synergy, HEK293 knockout cell line

## Abstract

Tyrosyl-DNA phosphodiesterase 1 (TDP1) catalyzes the cleavage of the phosphodiester bond between the tyrosine residue of topoisomerase 1 (TOP1) and the 3′ phosphate of DNA in the single-strand break generated by TOP1. TDP1 promotes the cleavage of the stable DNA–TOP1 complexes with the TOP1 inhibitor topotecan, which is a clinically used anticancer drug. This article reports the synthesis and study of usnic acid thioether and sulfoxide derivatives that efficiently suppress TDP1 activity, with IC_50_ values in the 1.4–25.2 μM range. The structure of the heterocyclic substituent introduced into the dibenzofuran core affects the TDP1 inhibitory efficiency of the compounds. A five-membered heterocyclic fragment was shown to be most pharmacophoric among the others. Sulfoxide derivatives were less cytotoxic than their thioester analogs. We observed an uncompetitive type of inhibition for the four most effective inhibitors of TDP1. The anticancer effect of TOP1 inhibitors can be enhanced by the simultaneous inhibition of PARP1, TDP1, and TDP2. Some of the compounds inhibited not only TDP1 but also TDP2 and/or PARP1, but at significantly higher concentration ranges than TDP1. Leader compound **10a** showed promising synergy on HeLa cells in conjunction with the TOP1 inhibitor topotecan.

## 1. Introduction

Genomic instability is one of the major driving forces of carcinogenesis. Studies of DNA repair mechanisms and their regulation are directly related to the search for the optimal ways of treating oncological and other human diseases. Chemotherapy is one of the main methods of treating various malignancy types. Anticancer chemotherapeutic drugs, by their principle of action, damage DNA in a targeted manner. They are powerful cell poisons that have a detrimental effect on the rapidly dividing cells of malignant tumors with a comparatively less negative damaging effect on the healthy, normally dividing cells and tissues of the organism. Although DNA repair is essential for a healthy cell, during anticancer therapy repair enzymes of the cancer cells counteract the efficacy of anticancer agents. Thus, DNA repair leads to a decrease in the effectiveness of therapy and contributes to the resistance of malignancies to chemotherapy. In recent years, much focus has been put on the DNA repair enzymes as targets for drug development. Researchers are actively searching for new compounds that suppress the DNA repair enzymes activity to increase the efficiency of anticancer therapy. Currently, tyrosyl-DNA phosphodiesterase 1 (TDP1) and poly(ADP-ribose) polymerase 1 (PARP1) are considered as promising target DNA repair enzymes for creating drugs [1,2].

TDP1 is involved in repairing stalled topoisomerase 1–DNA complexes by catalyzing the hydrolysis of the phosphodiester bond between the tyrosine residue of topoisomerase 1 (TOP1) and the 3’ phosphate of DNA in the single-strand break generated by TOP1. TDP1 also catalyzes the cleavage of phosphodiester bonds in other DNA–protein adducts and a number of different lesions at the 3’ end of DNA [3]. TDP1 plays a key role in removing the damage from DNA caused by the anticancer drugs used in clinical practice, such as topotecan (Tpc) and irinotecan, which are derivatives of the natural compound camptothecin [4,5]. Consequently, TDP1 activity may be a possible cause of tumor resistance to TOP1 inhibitors.

Tyrosyl-DNA phosphodiesterase 2 (TDP2) is a DNA repair enzyme that catalyzes the hydrolysis of dead-end complexes between DNA and the topoisomerase 2 (TOP2) active site tyrosine residue. TDP2 can remove a variety of covalent adducts from DNA through hydrolysis of a 5′ phosphodiester bond, giving rise to DNA with a free 5′ phosphate. TOP2 inhibitors stabilize the TOP2–DNA covalent complex and induce cell death [6]. TOP2 inhibitors (etoposide, doxorubicin) are widely used in clinical practice as antineoplastic drugs. TDP2 activity reduces the effectiveness of these drugs and, vice versa, TDP2 deficiency leads to a significant increase in sensitivity to TOP2 inhibitors [7,8]. The same as TDP1 inhibitors, TDP2 inhibitors can significantly increase the effectiveness of chemotherapy by synergizing with TOP2 inhibitors. The most effective TDP2 inhibitors today are deazaflavins [9,10]. Deazaflavin has been shown to synergize in vitro with etoposide at non-toxic concentrations [9]. It should be noted that deazaflavins have unsatisfactory pharmacokinetic characteristics, which makes it necessary to search for inhibitors of new structural types. Several TDP2 inhibitors of different chemical groups have already been proposed, but most of them had moderate efficacy [11]. Recently, though, some more efficient TDP2 inhibitors have been found [12,13,14]

TDP1 and TDP2 have little overlapping activity because TDP1 has a weak activity for 5′-phosphotyrosyl bonds, and TDP2 has a weak activity for 3′-phosphotyrosyl bonds [5]. Nevertheless, the ability of TDP1 and TDP2 to take on the functions of each other makes it highly promising to use the selective inhibitors of these two enzymes together, or to create agents capable of simultaneously inhibiting both TDP1 and TDP2. Simultaneous suppression of the activity of these two enzymes can be used to increase the effectiveness of a large set of clinically important anticancer drugs, TOP1 and TOP2 inhibitors. Recently, the first triple inhibitors of TOP1/TDP1/TDP2 have been discovered by Pommier’s group [15], which exhibit only moderate activity against TDP1 and weak activity against TDP2.

PARP1 catalyzes the synthesis of poly(ADP-ribose) (PAR), which is responsible for the post-translational modification of proteins, as an immediate DNA damage response of the cell. PARP1 is a member of various DNA repair pathways [16,17]. Inhibition of PARP1 activity makes it possible to sensitize tumor cells to the action of chemotherapeutic drugs. Olaparib was the first example of therapeutic synthetic lethality in oncology approved by the FDA for the treatment of advanced ovarian cancers associated with defective BRCA1/2 [18]. To date, the FDA has approved four different PARP inhibitors, olaparib (2014), rucaparib (2016), niraparib (2017), and talazoparib (2018), for the treatment of ovarian, fallopian tube, breast, and peritoneal cancers [19]. More inhibitors are in various stages of development and preclinical testing. In addition to cancer, PARP inhibitors are also promising in the treatment of cardiovascular diseases [20]. The anticancer effect of TOP1 inhibitors can be significantly enhanced by the simultaneous inhibition of PARP1 and TDP1. PARP1–TDP1 interplay was shown in a series of publications. PARylation of TDP1 enhances its recruitment to sites of DNA damage without interfering with the catalytic activity of TDP1 [21]. Briefly, it was shown [21] that the N-terminal domain of TDP1 directly binds the C-terminal domain of PARP1, and TDP1 is PARylated but not inactivated by PARP1. PARP1 is known to interact with TDP1 directly with K_D_ 120 nM [22]. PARP1 stimulates the enzymatic activity of TDP1 on AP sites [23]. TDP1 can repair the covalent DNA–PARP1 crosslink at the apurinic/apyrimidinic (AP) site in double-stranded DNA [24,25]. Other authors also noticed that TDP1 together with PARP1 inhibition could be a successful cancer treatment strategy [26,27]. TDP1 expression is correlated with other DNA repair genes, including PARP1, BRCA2, and BRCA1 [28]. The combination of 70% to 90% Tdp1 knockdown and 10 mmol/L of the PARP1 inhibitor rucaparib was found to reduce the proliferation of A204, Birch, RH30, and CW9019 cells more than either of these treatments alone [29]. These facts make urgent the search for dual inhibitors of TDP1 and PARP1. In the other work [30], TDP1, together with PARP1, were shown to be essential cellular proteins in cancerogenic [31] human papillomavirus HPV18 replication, thus making those proteins good targets for developing HPV inhibitors. The authors also noticed that TDP1 and PARP1 inhibitors might also be effective against HPV-induced cancer.

**Figure 1:**
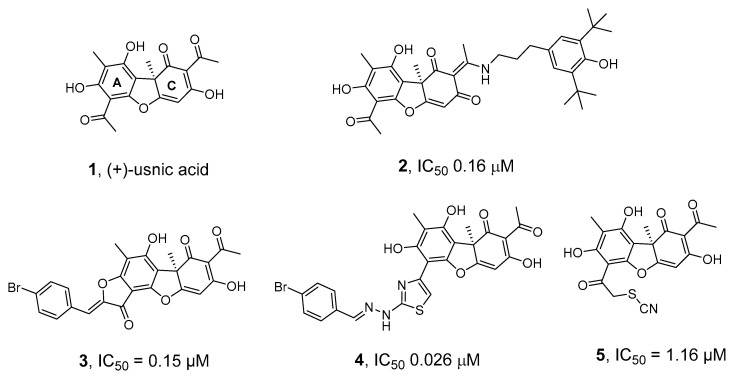
Structures of usnic acid (**1**) and its derivatives (**2**–**5**) that inhibit TDP1 [32,33,34,35,36].

We have previously discovered highly effective inhibitors of TDP1 based on the secondary metabolite of lichens usnic acid **1** (UA)—compounds **2**–**5** (Figure 1) [32,33,34,35,36]—the synergistic action of which, when combined with the TOP1 inhibitor Tpc, was confirmed in experiments on cell cultures and in animal models [36,37,38].

**Figure 2:**
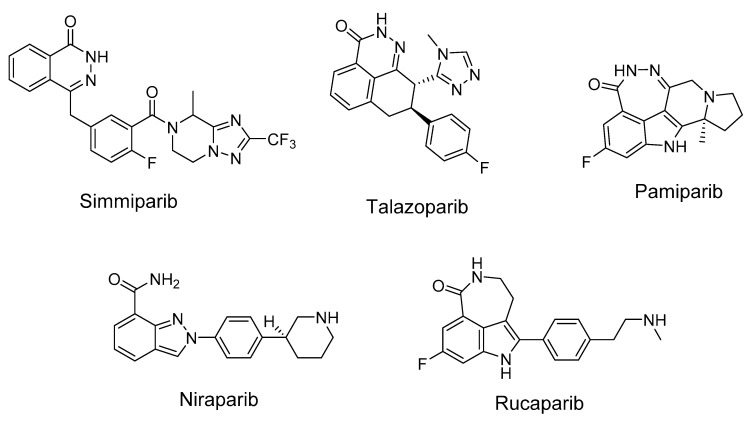
Some known inhibitors of PARP1.

We also previously studied the PARP1-inhibiting activity of various UA derivatives [39]. Most of the UA derivatives modified by the substituents in the C ring and the introduction of a fused ring or heterocycles into the A ring had low PARP1 affinity. The PARP1 inhibitory activity was facilitated by the introduction of aromatic substituents into the acyl fragment of the A ring of UA. Analysis of the literature showed that the pharmacophore fragments with respect to PARP1 are heterocycles containing one or several heteroatoms, primarily nitrogen (Figure 2) [40].

**Figure 3:**
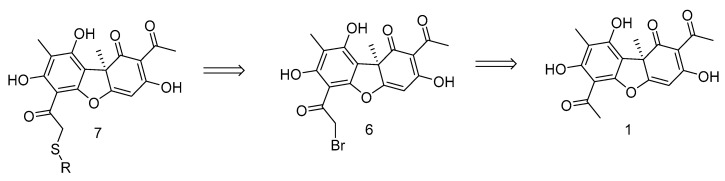
Retrosynthetic scheme for the synthesis of compound **7**.

We chose UA derivative **7** for the design of the joint TDP1 and PARP1 action inhibitors, which allow easy introduction of heteroatom-rich fragments into the acyl fragment of the ring A of UA (Figure 3). The activity of this type of UA derivative against DNA repair enzymes, including TDP1 and PARP1, has not been studied previously.

In this work, we propose to use an approach for the synthesis of potential TDP1 inhibitors or dual inhibitors based on the construction of a derivative from a UA backbone, a flexible linker, and a heterocycle to synthesize a set of compounds derivatized through cycle A. The aim of this study was to assess the inhibitory ability of the UA derivatives against three DNA repair enzymes, search for selective and multitarget agents, study the inhibition mechanism assessment of toxicity, and study the prospects of using these compounds as potential pharmacological agents of in anticancer therapy.

## 2. Results and Discussion

### 2.1. Chemistry

In order to find out the structure–activity relationship (SAR), we synthesized the set of usnic acid (UA) derivatives of **7**, with five- and hexa-membered mono- and bicyclic heterocyclic along with non-heterocyclic and acyclic substituents.

**Scheme 1:**
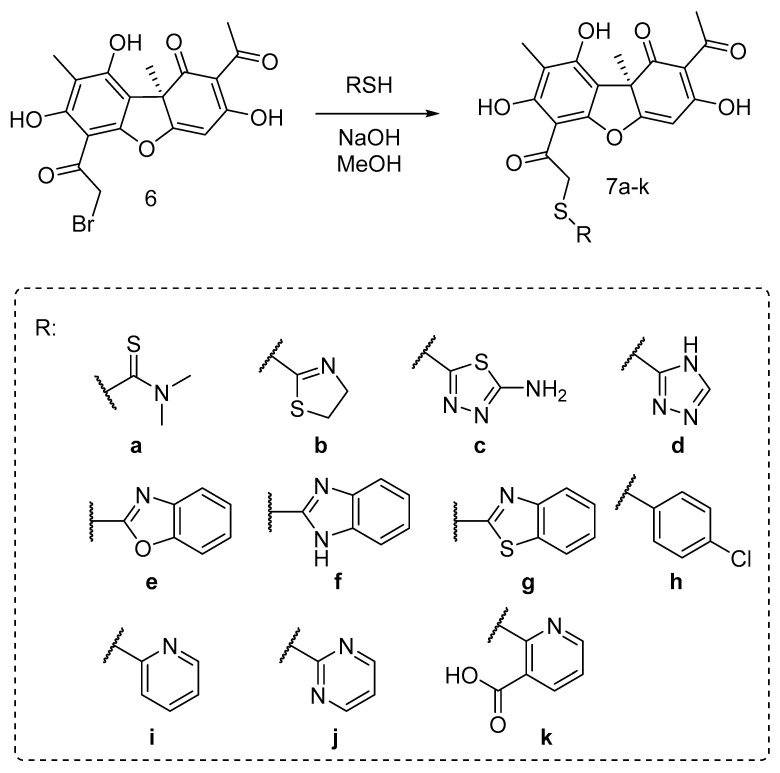
Synthesis of the thioether usnic acid derivatives.

The desired novel and known UA thioethers **7a**–**k** were synthesized using the procedure described early [41], by reaction of bromousnic acid **6** with the corresponding thiols in presence of NaOH. The target thioethers were obtained in 49–94% yield (Scheme 1).

**Scheme 2:**
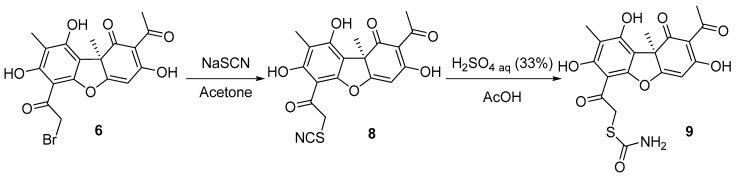
Synthesis of the thiocarbamate usnic acid derivative **9**.

UA derivative **9** was synthesized in two steps (Scheme 2). At first, the reaction of the bromousnic acid with sodium thiocyanate in acetone resulted in obtaining compound **8**, with a 95% yield. Then, UA thiocyanate derivative **8** was hydrolyzed. The hydrolysis was performed with (33%) sulfuric acid in glacial acetic acid to obtain the desired compound **9**, with a 38% yield.

**Scheme 3:**
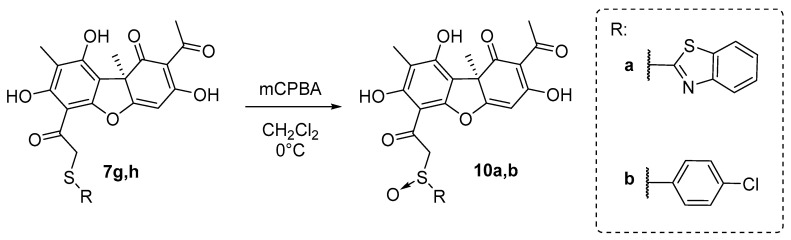
Synthesis of the sulfoxide usnic acid derivatives **10a**,**b**.

The target sulfoxide derivatives of **10** were obtained using the procedure described in [42], which is by a peroxidation reaction of the corresponding thioethers **7g**,**h** with mCPBA in CH_2_Cl_2_ at 0 °C (Scheme 3). The desired compounds **10a**,**b** were obtained, with 70–75% yields. 

### 2.2. Biology

#### 2.2.1. Real-Time Fluorescence Assay of TDP1 Activity

We tested UA and all 14 newly synthesized UA derivatives for their TDP1 inhibitory properties by measuring their IC_50_ values using a real-time fluorescent assay [43]. All 14 obtained compounds (Table 1) were shown to inhibit TDP1 in the low-enough concentration range (IC_50_ 1.7–25.2 μM). UA does not inhibit TDP1 in these concentrations. We can assume that the structure of the heterocyclic fragment bound to the sulfur atom affects the TDP1 inhibitory activity of the thioether derivatives. There is a five-membered heterocyclic fragment in the effective compounds (**7b**,**c**,**e**–**h** and **10a**,**b**), with an IC_50_ of 1.4–4.4 μM, except for **7d** (IC_50_ 25.2 μM), whereas the compounds containing a six-membered heterocycle (**7i**–**k**) inhibit TDP1 at higher concentrations (IC_50_ > 11 μM). It is possible that the presence of a 4-halophenyl substituent in the six-membered heterocycle (**7h** and **10b**, IC_50_ 2.2 μM and 1.4 μM, respectively) enhances the inhibitory properties of the compounds. Four of the most effective inhibitors of TDP1 were compounds **7g**,**h** and **10a**,**b** (IC_50_ of 1.7 μM, 2.2 μM, 2.1 μM, and 1.4 μM, respectively). Compounds **10a**,**b** are sulfoxide analogs of **7g**,**h**. 

#### 2.2.2. Type of Inhibition of TDP1 Enzyme Reaction for the Most Effective Compounds

The reversible enzyme inhibitors were classified as competitive, uncompetitive, non-competitive, or mixed, depending on the binding of the enzyme, to the enzyme–substrate complex (ES) or to the enzyme–substrate–inhibitor triple complex (ESI). The enzyme could bind the inhibitor at the different steps of catalysis (before or after the substrate) and at the substrate-binding site or another site. The effect of the inhibitor on enzymatic activity can be analyzed using graphical representations of the Michaelis–Menten equation. We defined the type of inhibition for the four most effective inhibitors of TDP1: **7g**,**h** and **10a**,**b** (IC_50_~1.4–2.2 μM, Table 1) by obtaining the dependence of the reaction rate on the substrate concentration and dependence of V_max_ and K_M_ on the concentration of the inhibitor under steady-state reaction conditions. For all four TDP1 inhibitors, we observed a fall in both the V_max_ and K_M_ values with an increasing concentration of the inhibitor, which is typical for the uncompetitive type of inhibition (Table S1, Figure S25) when the inhibitor binds the enzyme–substrate complex but does not bind free enzyme. Thus, it was determined that no studied inhibitors demonstrate the competitive mechanism of inhibition. TDP1 has a catalytic pocket with a narrow positively charged cleft where it binds DNA and with a relatively large cleft that contains a mixed charge distribution for the TOP1 fragment binding [44,45,46]. It is known that TDP1 hydrolyzes the DNA–TOP1 adducts via two coordinated nucleophilic attacks of two His residues (His263 and His493) with the covalent intermediate complex formation [45,46]. The enzyme reaction catalyzed by TDP1 could be inhibited in two steps: the first one is the inhibition of the TDP1 binding to the DNA substrate (the nucleophilic attack of His263). The second step is releasing TDP1 from the transition complex (nucleophilic attack by a water molecule activated by His493). Inhibition of the second step prevents the release of the DNA 3′-phosphate product from the DNA–TDP1 complex. Spinocerebellar ataxia with axonal neuropathy (SCAN1) is a neurological disease that is caused by a TDP1 His493Arg mutation. This mutation prevents the second step of hydrolysis of the intermediate complex and the enzyme remains covalently bound to DNA [47]. The uncompetitive inhibitors also prevent the second step of the reaction, stabilizing the enzyme–DNA covalent complex, which can lead to the accumulation of the single-strand breaks in the cell.

#### 2.2.3. The Effect of TDP1 Inhibitors on Cells Viability

**Figure 4:**
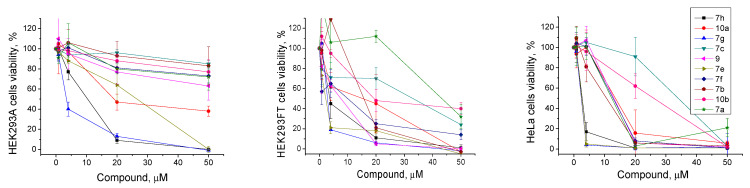
TDP1 inhibitors’ intrinsic cytotoxicity on HEK293A, HEK293FT, and HeLa cells, and the dose-dependent action of the most effective compounds. The values were normalized on the control wells with the cells in 1% DMSO.

The low intrinsic cytotoxicity of the additional drug component in the clinically used anticancer cocktails with traditional drugs is very important for treatment. This approach in chemotherapy can reduce the potential resulting toxic load on the body in the case of a combined action of these inhibitors on the activity of several targets. The aim of this part of the study was to demonstrate the ability of the found TDP1 inhibitors to enhance the cytotoxicity of the clinically used camptothecin derivative Tpc on the cancer cells. First, we analyzed the intrinsic cytotoxicity for 10 of the most effective TDP1 inhibitors (IC_50_ 1.4–6.6 μM, Table 1) on the HEK293A, HEK293FT, and HeLa cell lines by colorimetric tests. The effects of the tested compounds on cell survival are shown on Figure 4 and the CC_50_ values for three cell lines are shown in Table 1. The intrinsic cytotoxicity of a number of the studied compounds is quite high, namely, they have close IC_50_ values against the TDP1 and CC_50_ values on one or several studied cell lines. Most of the compounds generally were more toxic for the HEK293FT and HeLa cell lines than for the HEK293A cell line (Table 1 and Figure 4). The cytotoxicity of the three leader compounds, **7g**, **10a**, and **7h**, was quite high. Interestingly, the sulfoxide analogs **10a**,**b** were less cytotoxic than their thioester analogs **7g**,**h**. Compound **10b** was one of the least toxic among the ten tested compounds.

#### 2.2.4. PARP1 and PARP2 Activity in the Presence of TDP1 Inhibitors

The next step of our study was to test the inhibitors for their ability to inhibit PARP1 and PARP2. UA and the 14 synthesized compounds were tested for the suppression of the ability of PARP1 and PARP2 to include a radioactive label into the acid-insoluble reaction product, poly(ADP-ribose) (PAR). None of the tested compounds inhibited PARP2 in the applied reaction conditions. All the results obtained for PARP1 inhibition are shown in Table 1. Eight compounds exhibited a weak inhibitory activity against PARP1 (40–80% of residual activity) at a 1 mM concentration, working at significantly higher concentration ranges than against TDP1. The best effect we observed was for **9**; the residual activity was 40 ± 5% under the reaction conditions (0.5 mM concentration of the compound), indicated in Materials and Methods. For PARP1, compounds with smaller substituents, an aliphatic or benzene moiety, had a higher inhibitory effect.

#### 2.2.5. TDP2 Activity in the Presence of TDP1 Inhibitors

To study the ability of the investigated compounds to inhibit TDP2 activity, we separated the TDP2 reaction products under denaturing conditions in polyacrylamide gel (PAGE). We tested the ability of TDP2 to eliminate tyrosine residues from the 5’ end of the oligonucleotide substrate in the absence and presence of TDP1 inhibitors. UA did not inhibit TDP2 (Table 1, Figure S26). All 14 newly synthesized UA derivatives inhibited TDP2 at a 1 μM concentration, but almost did not inhibit its activity at a 0.200 mM concentration. The residual TDP2 activity for the best four TDP1 inhibitors, **7g**,**h** and **10a**,**b**, was from 30 to 60%, depending on the tested compound, at a 1 mM concentration of the inhibitor. We tested the ability of compounds **7g**,**h** and **10a**,**b** to inhibit TDP2 activity at 0.2 mM and 0.5 mM concentrations (Figure S26). All four tested compounds inhibited TDP2 insignificantly, at higher concentration ranges than TDP1. Interestingly, the best TDP1 inhibitors, **7g**,**h** and **10a**, exhibited the highest effect on TDP2.

#### 2.2.6. The Effect of TDP1 Inhibitors on Cell Viability in Combination with Topotecan

TDP1 is able to cleave the TOP1–DNA complex, thus preventing the action of Tpc and decreasing the efficiency of this clinically used drug. The inhibition of TDP1 activity can increase the efficiency of Tpc. Next, we checked the cytotoxic effect of the combination of Tpc and examined the TDP1 inhibitors compared to Tpc alone on HeLa cells and HEK293 cells. 

**Figure 5:**
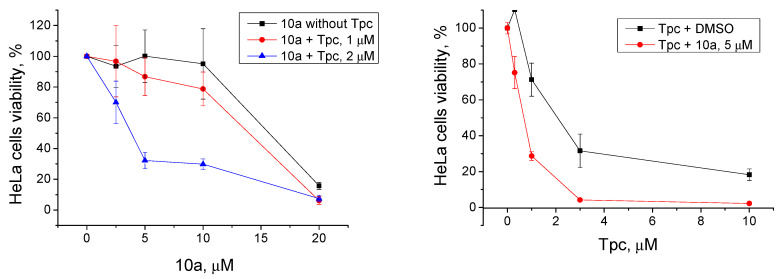
Left panel: The influence of Tpc at 1 μM and 2 μM concentrations on **10a** cytotoxicity. B: The influence of the **10a** derivative at a 5 μM concentration on Table 1. DMSO, for the red curve on the cells treated with 1 μM topotecan and 1% DMSO, and for the blue curve on the cells treated with 2 μM topotecan and 1% DMSO, in order to exclude the effect of topotecan itself and see the effect of the drug combination. Right panel: cells treated with 1% DMSO (black line) or 5 μM **10a** (red line) were taken as 100% in order to evaluate the effect of the combination rather than the individual substance **10a**.

Only one compound, **10a**, of the most effective TDP1 inhibitors (IC_50_ 2.1 μM, Table 1) showed promising synergy on HeLa cells in conjunction with Tpc. We observed the suppressed cell growth in the joint presence of the TDP1 inhibitor and Tpc on HeLa cells compared to Tpc alone (Figure 5). We observed the synergistic effect both when we titrated **10a** with addition of fixed Tpc (1 μM or 2 μM) and vice versa when we titrated Tpc with addition of fixed **10a** (5 μM). The CC_50_ value of **10a** decreased from 15 μM (only **10a**) to 3 μM (**10a** with 2 μM Tpc). The CC_50_ value of Tpc decreased from 2 μM (only Tpc) to 0.7 μM (Tpc with 5 μM **10a**).

**Figure 6:**
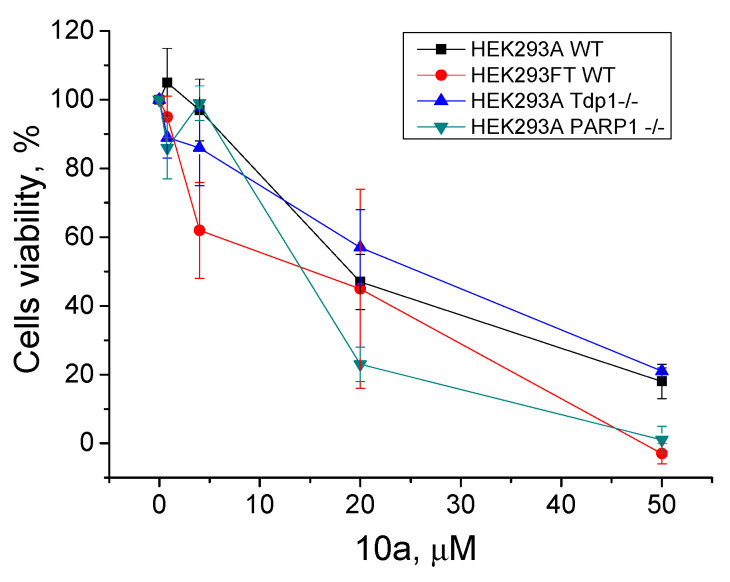
Intrinsic cytotoxicity of **10a** on HEK293FT and HEK293A cells, as well as on the Tdp1 and *PARP1* knockout HEK293A cell lines (HEK293A *Tdp1*-/- and HEK293A *PARP1*-/-); the dose-dependent action. The values were normalized to the control wells with the cells in 1% DMSO.

In our previous works, we checked the cytotoxic effect of Tpc and the TDP1 inhibitors—monoterpene 3-carene-derived compounds [48,49] and UA combined with monoterpenoids [49], measured separately and jointly with Tpc—using a panel of HEK293FT and HEK293A *Tdp1* knockout isogenic clones. We showed that *Tdp1* knockout cells were more sensitive to Tpc compared to WT cells. The data on the HEK293FT mutants were of low reproducibility [48], which is why we decided to change the basic cell line to HEK293A [49]. In this work, we created new *PARP1*-/- HEK293A cells using the CRISPR–Cas9 approach (Figure S27). The sensitivity of the *PARP1*-/- cell line to Tpc was lower than WT HEK293A cells (CC_50_ 50 ± 5 nM and 27 ± 4 nM respectively, Figure S28). We checked the intrinsic cytotoxicity of the leader compound (**10a**) on the HEK293 wild type and *TDP1*-/- and *PARP1*-/- cell lines using a colorimetric test (Figure 6). The cytotoxicity of **10a** was nearly the same for all the cell lines, with a CC_50_ of 15–20 μM (Figure 6). We did not observe any effect of the joint presence of **10a** and Tpc on any of these cells (data not shown). There was no difference between the WT and mutant cells.

## 3. Materials and Methods

### 3.1. Chemistry

The analytical and spectral studies were conducted in the Chemical Service Center for the collective use of the Siberian Branch of the Russian Academy of Sciences. PMR and ^13^C NMR spectra were recorded in CDCl_3_ or DMSO-d_6_ using solvent resonances (^1^H 7.24 ppm, ^13^C 76.90 ppm, and ^1^H 2.50 ppm, ^13^C 39.50, respectively) as the standards on a Bruker AV-400 spectrometer (Bruker Corporation, Germany; operating frequencies 400.13 MHz for ^1^H and 100.61, for ^13^C). Mass spectra (ionizing-electron energy 70 eV) were measured with a DFS Thermo Scientific high-resolution mass spectrometer (Thermo Fisher Scientific, Waltham, MA, USA). Macherey–Nagel silica gel (63–200 μ) was used for the column chromatography. Thin-layer chromatography was performed on TLC Silica gel 60 plates (UV-254, Merck, Darmstadt, Germany) (Figures S1–S24). 

(+)-UA {**1**, [α]_D_ +478° (*c* 0.1, CHCl_3_)} was isolated from a mixture of *Usnea* lichen species using the literature method [50]. Bromousnic acid **2** was synthesized according to the literature method [51]. Usnic acid derivative **9** was synthesized according to the literature [35]. 

Synthetic starting materials, reagents, and solvents were purchased from Sigma-Aldrich (St. Louis, MO, USA), Acros Organics (Geel, Belgium), and AlfaAesar (Heysham, UK) (95–99% pure). All chemicals were used as described unless otherwise noted. Reagent-grade solvents were redistilled prior to use.

#### 3.1.1. Reaction of 2 with Thiols (General Method)

A weighed portion of KOH (1.1 mmol), MeOH (6 mL) and the appropriate thiol (1.1 mmol) were placed into a flask, stirred at room temperature for 10–15 min, treated with a solution of **2** (1 mmol) in CH_2_Cl_2_ (2 mL), stirred at room temperature for 2–3 h until the reaction was finished (TLC monitoring), washed with distilled H2O (two times the volume), dried over MgSO_4_, and concentrated. If necessary, the solid was chromatographed over silica gel using a CH_2_Cl_2_ eluent.

*(2R)-4-acetyl-10-{2-[(dimethylcarbamothioyl)sulfanyl]acetyl}-5,11,13-trihydroxy-2,12-dimethyl-8-oxatricyclo [7.4.0.0^2,7^]trideca-1(13),4,6,9,11-pentaen-3-one 7a*

Yellow amorphous powder, yield 94%. ^1^H NMR (CDCl_3_, δ) 1.77 (3H, s), 2.09 (3H, s), 2.65 (3H, s), 3.46 (3H, s), 3.54 (3H, s), 4.92 (2H, m), 6.02 (1H, s), 11.11 (OH, s), 12.81 (OH, s), 18.84 (OH, s). ^13^C NMR (CDCl_3_, δ): 7.4, 27.7, 31.9, 41.5, 45.6, 47.9, 58.8, 98.5, 100.9, 104.1, 105.0, 109.3, 154.7, 157.7, 163.5, 178.8, 191.4, 194.5, 195.2, 197.8, 201.6. HRMS m/z 463.0750 (calcd for C_21_H_21_O_7_N_2_^32^S_2_, 463.0754).

*(2R)-4-acetyl-10-[2-(4,5-dihydro-1,3-thiazol-2-ylsulfanyl)acetyl]-5,11,13-trihydroxy-2,12-dimethyl-8-oxatricyclo[7.4.0.0^2,7^]trideca-1(13),4,6,9,11-pentaen-3-one 7b*

Yellow amorphous powder, yield 49%. ^1^H NMR (CDCl_3_, δ) 1.73 (3H, s), 2.06 (3H, s), 2.63 (3H, s), 3.41 (2H, bt), 4.15 (2H, bt), 4.43 (2H, m), 5.99 (1H, s), 11.09 (OH, s), 12.71 (OH, s), 18.81 (OH, s). ^13^C NMR (CDCl_3_, δ): 7.5, 27.8, 31.9, 43.0, 58.8, 63.7, 75.7, 98.7, 100.2, 104.1, 105.1, 109.5, 154.6, 157.9, 163.7, 167.7, 178.7, 191.5, 194.2, 197.8, 201.7. HRMS m/z 461.0592 (calcd for C_21_H_19_O_7_N^32^S_2_, 461.0598).

*(2R)-4-acetyl-10-{2-[(5-amino-1,3,4-thiadiazol-2-yl)sulfanyl]acetyl}-5,11,13-trihydroxy-2,12-dimethyl-8-oxatricyclo[7.4.0.* *0^2,7^]trideca-1(13),4,6,9,11-pentaen-3-one 7c*

Yellow amorphous powder, yield 81% ^1^H NMR (DMSO-d_6_, δ) 1.71 (3H, s), 1.99 (3H, s), 2.56 (3H, s), 4.62 (2H, s), 6.89 (1H, s), 7.32 (NH2, s), 11.56 (OH, bs), 12.75 (OH, s). ^13^C NMR (DMSO-d_6_, δ): 7.6, 27.9, 31.5, 44.2, 58.3, 98.7, 100.0, 105.1, 105.6, 107.5, 107.6, 148.7, 154.8, 157.2, 157.4, 162.3, 162.6, 170.2, 178.3, 190.9, 195.4, 197.4, 201.0. HRMS m/z 475.0507 (calcd for C_20_H_17_O_7_N_3_^32^S_2_, 475.0502).

*(2R)-4-acetyl-10-[2-(1H-1,3-benzodiazol-2-ylsulfanyl)acetyl]-5,11,13-trihydroxy-2,12-dimethyl-8-oxatricyclo[7.4.0.0^2,7^]trideca-1(13),4,6,9,11-pentaen-3-one 7f*

Yellow amorphous powder, yield 54%. ^1^H NMR (CDCl_3_, δ) 1.70 (3H, s), 2.07 (3H, s), 2.64 (3H, s), 4.67 (2H, m), 5.94 (1H, s), 7.16 (2H, m), 7.47 (2H, m), 11.18 (OH, s), 12.64 (OH, bs), 18.78 (OH, bs). ^13^C NMR (CDCl_3_, δ): 7.3, 27.7, 31.7, 42.9, 58.5, 98.6, 100.0, 104.1, 104.9, 109.1, 113.8, 122.1, 138.4, 148.5, 154.5, 157.9, 163.3, 178.3, 191.3, 194.5, 197.6, 201.5. HRMS m/z 492.0981 (calcd for C_25_H_20_O_7_N_2_^32^S, 492.0986).

*(2R)-4-acetyl-5,11,13-trihydroxy-2,12-dimethyl-10-[2-(pyridin-2-ylsulfanyl)acetyl]-8-oxatricyclo[7.4.0.* *0^2,7^]trideca-1(13),4,6,9,11-pentaen-3-one 7i*

Yellow amorphous powder, yield 90% ^1^H NMR (CDCl_3_, δ) 1.76 (3H, s), 2.09 (3H, s), 2.65 (3H, s), 4.70 (2H, m), 5.92 (1H, s), 6.98 (1H, dd, J_1_ = 7.3 Hz, J_2_ = 5.0 Hz), 7.26 (1H, d, J = 7.3 Hz), 7.49 (1H, dt, J_1_ = 7.3 Hz, J_2_ = 1.5 Hz), 8.33 (1H, d, J = 5.0 Hz), 11.09 (OH, s), 12.86 (OH, s), 18.83 (OH, s). ^13^C NMR (CDCl_3_, δ): 7.5, 27.8, 32.0, 40.3, 58.9, 98.5, 100.5, 104.1, 105.1, 109.5, 119.8, 122.0, 136.1, 149.3, 154.7, 156.8, 157.7, 163.8, 179.0, 191.6, 195.9, 197.9, 201.7. HRMS m/z 453.0874 (calcd for C_23_H_19_O_7_N^32^S, 453.0877).

*(2R)-4-acetyl-5,11,13-trihydroxy-2,12-dimethyl-10-[2-(pyrimidin-2-ylsulfanyl)acetyl]-8-oxatricyclo[7.4.0.* *0^2,7^]trideca-1(13),4,6,9,11-pentaen-3-one 7j*

Yellow amorphous powder, yield 92% ^1^H NMR (CDCl_3_, δ) 1.77 (3H, s), 2.09 (3H, s), 2.65 (3H, s), 4.65 (2H, m), 5.98 (1H, s), 6.96 (1H, t, J = 6.4 Hz), 8.45 (2H, d, J = 6.4 Hz), 11.09 (OH, s), 12.82 (OH, s), 18.83 (OH, s). ^13^C NMR (CDCl_3_, δ): 7.5, 27.8, 32.0, 41.4, 58.9, 98.5, 100.5, 104.1, 105.1, 109.5, 116.77, 154.7, 157.2, 157.4, 163.7, 170.7, 178.9, 191.5, 195.2, 197.8, 201.7. HRMS m/z 454.0823 (calcd for C_22_H_18_O_7_N_2_^32^S, 454.0829).

#### 3.1.2. Hydrolysis of Compound **8**

Compound **8** (1 mmol) was dissolved in glacial acetic acid (15 mL). The sulfuric acid water solution (30%, 0.5 mL) was added and the mixture was stirred for 4 h. After that, the resulted mixture was treated with water and the precipitate that formed was filtered off and dried in air. The solid was chromatographed over silica gel using a CH_2_Cl_2_ eluent with an MeOH gradient.

*(2R)-4-acetyl-10-[2-(carbamoylsulfanyl)acetyl]-5,11,13-trihydroxy-2,12-dimethyl-8-oxatricyclo[7.4.0.0^2,7^]trideca-1(13),4,6,9,11-pentaen-3-one 8*

Yellow amorphous powder, yield 38%. ^1^H NMR (CDCl_3_, δ) 1.74 (3H, s), 2.07 (3H, s), 2.64 (3H, s), 4.43 (2H, m), 5.80 (NH2, bs) 6.00 (1H, s), 11.12 (OH, s), 12.76 (OH, s), 18.82 (OH, s). ^13^C NMR (CDCl_3_, δ): 7.5, 27.8, 31.9, 40.8, 58.8, 98.7, 100.2, 104.2, 105.1, 109.5, 154.7, 158.0, 163.7, 167.7, 178.7, 191.5, 194.9, 197.8, 201.7. HRMS m/z 419.0671 (calcd for C_19_H_17_O_8_N^32^S, 419.0669).

#### 3.1.3. Oxidation of Compounds **7g**,**h**

Thioether **7g** or **7h** (1 mmol) was dissolved in methylene chloride (5 mL) on an ice bath. Meta-chloroperoxybenzoic acid (3 mmol) was added and the resulted solution was stirred for 30 min at 0 °C. After that, the mixture was treated with a saturated sodium sulfite solution (3 mL) and the resulted mixture was stirred for 1 h at room temperature. Finally, the mixture was extracted with methylene chloride, dried over magnesium sulfate, and evaporated.

*(2R)-4-acetyl-10-[2-(1,3-benzothiazole-2-sulfinyl)acetyl]-5,11,13-trihydroxy-2,12-dimethyl-8-oxatricyclo[7.4.0.0^2,7^]trideca-1(13),4,6,9,11-pentaen-3-one 10a*

Yellow amorphous powder, yield 75%. ^1^H NMR (CDCl_3_, δ) 1.64 and 1.69 (3H, s), 2.06 and 2.09 (3H, s), 2.63 and 2.63 (3H, s), 4.72 and 5.01 (2H, m), 5.93 and 5.98 (1H, s), 7.46 (2H, m), 8.00 (2H, m), 11.18 and 11.21 (OH, s), 12.52 and 12.59 (OH, s), 18.78 (OH, bs). ^13^C NMR (CDCl_3_, δ): 7.4 and 7.4, 27.7, 31.7 and 31.8, 58.5 and 58.6, 68.0 and 68.6, 99.0, 101.3 and 101.4, 104.4 and 104.5, 105.0 and 105.1, 109.5 and 109.6, 122.1 and 122.2, 123.9 and 124.0, 126.4 and 127.0, 136.0 and 136.1, 153.5, 154.5 and 154.5, 158.8 and 158.9, 163.8 and 164.0, 176.1 and 176.2, 178.1, 190.4 and 190.6, 191.4, 197.6, 201.6 and 201.7.

### 3.2. Biology

#### 3.2.1. Preparation of Labeled Oligonucleotides

A single-stranded oligodeoxynucleotide 5’-FAM-AAC GTC AGG GTC TTC C-BHQ-1-3′ containing a 6-carboxamido fluorescein (6-FAM) fluorophore at the 5′-end and a Black Hole Quencher 1 (BHQ-1) at the 3′-end was employed as an internally-quenched probe for real-time detection of TDP1 activity [43]. Another single-stranded oligonucleotide 5′-Tyr-AAC GTC AGG GTC TTC C-FAM-3′ containing a 6-FAM label at the 3′-end and an L-tyrosine residue attached via the phenolic OH group to the 5′-terminal phosphate was used as a substrate for detection of TDP2 activity (Tyr = HOOC-CH(NH_2_)-CH_2_-C_6_H_4_-*p*-O~).

Oligonucleotides were synthesized essentially as described in a previous paper [33]. Briefly, both oligonucleotides were assembled on a Biosset ASM-800 automated DNA/RNA synthesizer (Novosibirsk, Russia) on 200 nmol scale by β-cyanoethyl phosphoramidite chemistry. The doubly labeled probe was prepared from standard 5′-DMTr-deoxynucleoside 3′-phosphoramidites, 6-FAM phosphoramidite for 5′-labeling, and a BHQ-1 CPG support for attachment of a Black Hole Quencher^TM^ BHQ-1 (all from Glen Research, Sterling, VA, USA), deprotected and purified under standard conditions as described above [33].

Synthesis of a 5′-tyrosinyl oligodeoxynucleotide substrate 5′-Tyr-AAC GTC AGG GTC TTC C-FAM-3’ was carried out on a specially prepared CPG 500 Å support containing *N*^α^-Fmoc-L-tyrosine with an unprotected phenol group esterified via the carboxy group to the 5’-OH group of a thymidine residue linked to the support through the 3′-OH by a succinate linker. Synthesis of the tyrosine support was accomplished as described previously [33]. In contradistinction to the previous paper, in this work the solid-phase DNA assembly was carried out with ‘reversed’ 3′-DMTr-deoxynucleoside 5′-phosphoramidites (ChemGenes, Wilmington, MA, USA) in the 5′→3′ direction using a free phenolic OH of the support-bound tyrosine as an anchoring point to introduce the tyrosine residue onto the 5′-end. 6-FAM phosphoramidite (Glen Research, Sterling, VA, USA) was coupled at the last cycle of the synthesis to place the label at the 3′-end. The oligonucleotide with a phosphotyrosine residue was cleaved from support by alkaline hydrolysis of the ester linkages (100 μL of 0.1 M NaOH, 1 h at 25 °C). Excess NaOH was quenched by ca. 50 μL of Amberlyst cation exchange resin beads in NH_4_^+^ form. The solid support was discarded, the supernatant and aqueous washings (2 × 100 μL) were combined, evaporated to dryness, and treated with 200 μL of a conc. (ca. 28%) aqueous NH_3_ solution at 55 °C for 16 h to remove all the remaining *N*-protecting groups from oligonucleotide. The DNA substrate was isolated, purified, and analyzed as described previously [33]: [M-H]^−^ calc. 5666.83, found 5666.90.

Human recombinant tyrosyl-DNA phosphodiesterase 1 (TDP1) and human recombinant poly(ADP-ribose)-polymerase1 (PARP1) were expressed in the *E. coli* system and purified as described [52,53].

#### 3.2.2. Expression and Purification of Human Recombinant Tyrosyl-DNA Phosphodiesterase 2 (TDP2)

The recombinant N-terminally His-tagged TDP2 was expressed in *E. coli* BL21 (DE3) cells. The plasmid pLATE-31-TDP2 expression vector was constructed as follows. cDNA encoding the TDP2 protein was cloned in the pLATE-31 expression vector using a LICator LIC Cloning and Expression System (Thermo Scientific, Waltham, MA,, USA). Using specific primers and total cDNA of HeLa cells, the TDP2 coding sequence was amplified by PCR and annealed with a linearized pLATE-31 vector. The sequence of the cloned cDNA was confirmed at the SB RAS Genomics Core Facility (ICBFM SB RAS, Novosibirsk, Russia). Plasmids were transformed into BL21 cells by electroporation, and the cells were grown in LB medium at pH 7.5 with 100 mg/mL ampicillin at 30 °C. Two hours after induction with 1 mM IPTG, cells were harvested. Cell pellets were thawed on ice, resuspended in binding buffer (0.5 M NaCl, 5% glycerol, 50 mM Tris-HCl, pH 8.0, and a mixture of protease inhibitors), and broken by sonication. After centrifugation, 10 mM imidazole was added to the supernatant. The Ni sepharose column (GE Healthcare, UK) was washed with binding buffer (0.5 M NaCl, 10 mM imidazole, 50 mM Tris-HCl, and pH 8.0). Elution of the proteins was carried out with an elution buffer (0.5 M NaCl, 500 mM imidazole, 50 mM Tris-HCl, pH 8.0, and protease inhibitors), and the eluate was loaded into the heparin sepharose column (GE Healthcare, Pollards Wood, UK). Elution of the proteins was carried out with a NaCl gradient of 0.1–1 M in 50 mM Tris-HCl, pH 8.0, with protease inhibitors. The protein TDP2 was stored in 50 mM NaCl, 50 mM Tris-HCl pH 8.0, 1 mM EDTA, 2 mM DTT, and 50% glycerol at −20 °C. The enzyme samples were estimated to be more than 95% pure. Enzyme concentrations were estimated by Bradford assay. The Coomassie-stained protein gel is shown in Supplementary Material (Figure S29).

#### 3.2.3. Real-Time Detection of TDP1 Activity

The biosensor (5′-[FAM] AAC GTC AGGGTC TTC C [BHQ]-3′) was synthesized in the Laboratory of Nucleic Acid Chemistry at the Institute of Chemical Biology and Fundamental Medicine (Novosibirsk, Russia) and was used for TDP1 enzyme activity real-time fluorescence detection [43]. The reaction mixture (200 μL) contained a TDP1 reaction buffer (50 mM Tris-HCl, pH 8.0, 50 mM NaCl, and 7 mM β-mercaptoethanol), 50 nM oligonucleotide, varied concentrations of the tested compounds, and purified TDP1 in a final concentration 1.5 nM. The reaction mixtures were incubated at a constant temperature of 26 °C in a POLARstar OPTIMA fluorimeter (BMG LABTECH, GmbH, Ortenberg, Germany). Fluorescence intensity was measured (Ex485/Em520 nm) every 1 min for 10 min. The average values of the half maximal inhibitory concentration (IC_50_) were determined using an eleven-point concentration response curve and calculated using MARS Data Analysis 2.0 (BMG LABTECH). The 50% inhibitory concentration (IC_50_) was defined as the concentration of the compound that inhibited 50% of the enzyme activity when compared to the untreated controls. At least three independent experiments were carried out to obtain the IC_50_ values. To determine the kinetic parameters of the TDP1 enzymatic reaction, the apparent maximum rate of enzymatic reaction (V_max_), Michaelis constant (K_M_), possible inhibition mechanism, and steady-state kinetic experiments were carried out at 5 fixed concentrations of the substrate, with variation in the inhibitor concentrations [54]. The standard reaction mixtures (200 μL) contained reaction buffer components; 50 nM, 100 nM, 200 nM, 500 nM, or 1000 nM substrate; an inhibitor; and 1.5 nM recombinant human TDP1. The initial kinetic curves were obtained in three independent experiments and statistically processed in OriginPro 8.6.0 (OriginLab, Northampton, MA, USA).

#### 3.2.4. Gel-Based TDP2 Activity Assay

Oligonucleotide 5′- tyrosine -AAC GTC AGG GTC TTC C- FAM -3′ was synthesized as described above and used for the indication of TDP2 enzyme activity in polyacrylamide gel. TDP2 gel-based assays were performed to a final volume 20 μL using 100 nM substrate incubated with 200 nM recombinant human TDP2 in the absence or presence of an inhibitor for 10 min at 37 °C in a buffer containing 50 mM Tris-HCl, pH 8.0, 50 mM NaCl, 7 mM β-mercaptoethanol, and BSA. Reactions were terminated by the addition of a gel loading buffer (TBE, 10% formamide, 7 M carbamide, 0.1% xylene cyanol, and 0.1% bromophenol blue, and 20 mM EDTA). The samples were heated before loading at 90 °C for 7 min. The reaction products were separated by electrophoresis in a 20% denaturing PAGE with 7 M carbamide at a ratio of acrylamide to bisacrylamide of 19:1. A Typhoon FLA 9500 phosphorimager (GE Healthcare, Boston, MA, USA) was used for gel scanning and imaging, and the data were analyzed with QuantityOne 4.6.7 software (Bio-Rad Laboratories, Inc., Hercules, CA, USA).

#### 3.2.5. PARP1 and PARP2 Enzyme Assay

The radioactive labeled [32P]-NAD+ was synthesized from α–[32P]-ATP according to [55]. The reaction of autopoly(ADP-ribosyl)ation was carried out as follows: for PARP1, 50 mM Tris-HCl, pH 8.0, 10 mM MgCl_2_, 150 mM NaCl, and 7 mM β-mercaptoethanol, as well as activated DNA 2 oe/mL, 0.3 mM [^32^P]-NAD+ at 37 °C. The reaction was initiated by adding PARP1 to 200 nM and the reaction mixtures were incubated for 2 min. For PARP2: 50 mM Tris-HCl, pH 8.0, 3 mM spermin, 150 mM NaCl, and 7 mM β-mercaptoethanol, as well as activated DNA 2 oe/mL, 0.6 mM [^32^P] NAD+ at 37 °C. The reaction was initiated by adding PARP2 to 800 nM and the reaction mixtures were incubated for 5 min. The tested compounds were added at a final concentration 500 nM for reactions with PARP1 and 1 μM for reactions with PARP2. The reaction was stopped by placing 10 µL aliquots onto Whatman 1 paper filters soaked with 5% TCA. The filters were washed with 5% TCA four times and dried in the air after the removal of TCA with 90% ethanol. The incorporation of radioactivity into the product was calculated using a Typhoon FLA 9500 scanner (GE Healthcare, Chicago, IL, USA). Measurements were done in at least two independent experiments.

#### 3.2.6. Cell Culture Cytotoxicity Assay

Cytotoxicity of the compounds was examined against human cell lines HEK293A (human embryonic kidney)—WT, TDP1 deficient (*Tdp1*-/-), PARP1 deficient (*PARP1*-/-), and HeLa (cervical cancer) using an EZ4U colorimetric test (Biomedica, Vienna, Austria). The HEK293A cell line was obtained from Thermo Fisher Scientific (Waltham, MA, USA), and the HeLa cell line was obtained from the Russian Cell Culture Collection (RCCC) Institute of Cytology RAS, St. Petersburg, Russia. The cells were grown in DMEM/F12 medium (Gibco, Thermo Fisher Scientific, Waltham, MA, USA), with 1x GlutaMAX (Gibco, Thermo Fisher Scientific, Waltham, MA, USA), 50 IU/mL penicillin, 50 μg/mL streptomycin (MP Biomedicals), and in the presence of 10% fetal bovine serum (Biolot, Saint-Petersburg, Russia) in a 5% CO_2_ atmosphere. Cells were grown in the presence of 1% DMSO in the control wells. After the formation of a 30–50% monolayer, the tested compounds were added to the medium, and the cell culture was monitored for 3 days. The values were normalized to their own control in each case. At least three independent experiments were carried out. The 50% cytotoxic concentration (CC_50_) was defined as the compound concentration that reduced the cell viability by 50% when compared to the untreated controls. The compound concentration that caused 50% cell growth inhibition was determined using OriginPro 8.6.0 software (OriginLab, Northampton, MA, USA). The measurements were carried out in three independent experiments.

#### 3.2.7. Plasmid Construction for Human *PARP1* Gene Knockout

sgRNAs design was performed using the Benchling CRISPR tool (https://www.benchling.com/, accessed on 9 November 2019). Two protospacers (PAM sequences in brackets) were selected for the DNA sequence deletion that includes 3–5 exons of the *PARP1* gene: PARP1-gRNA1 CTAGAACCTCCAATACCATG (TGG) and PARP1-gRNA2 GCAAGTGACCACAAAGGTGC (AGG). Corresponding oligonucleotides were cloned in plasmid pSpCas9(BB)-2A-GFP (PX458) (the plasmid was a gift from Feng Zhang (Addgene plasmid #48138; http://n2t.net/addgene:48138; RRID:Addgene_48138)) as previously described [48]. Transfection-grade plasmid DNA was isolated using the Plasmid Plus Midi Kit (QIAGEN, Hilden, Germany).

#### 3.2.8. Knockout HEK293A Clones’ Generation

5 × 10^5^ HEK293A cells were plated into each well of a 12-well plate and co-transfected with the constructed plasmids PARP1-gRNA1 and PARP1-gRNA2 (0.25 µg of each) using a Lipofectamine 3000 Reagent (Thermo Fisher Scientific, Waltham, MA, USA). The growth medium contained DMEM/F12 (Gibco) 1:1, 10% FBS (Gibco), 100 U/mL penicillin–streptomycin (Gibco, Thermo Fisher Scientific, Waltham, MA, USA), and 1× GlutaMAX (Gibco, Thermo Fisher Scientific, Waltham, MA, USA). A total of 48 h after transfection the cells were detached using TrypLE Express (TrypLE, Gibco, Thermo Fisher Scientific, Waltham, MA, USA), and the GFP-positive cell population was enriched by cell sorting using the BD FACSAria III Cell Sorter (BD Biosciences, Franklin Lakes, NJ, USA). Transfected cells were plated onto a 96-well plate, one cell per well. Single-cell clones grew for two weeks before they were replicated to another 96-well plate, so we obtained two equal 96-well plates with cell clones: one plate was used for PCR analysis of the deletion in the *PARP1* gene, and the other plate was used for the mutant cell clone multiplying.

#### 3.2.9. Analysis of CRISPR/Cas9-Mediated Deletions in *PARP1* Gene

Genome DNA was extracted from cells on one of two 96-well plates using 50 μL of QuickExtract™ DNA Extraction Solution (Lucigen, Madison, WI, USA) per well. The DNA extracts were diluted with 200 μL of mQ water. Two microliters of the diluted DNA extract were used to PCR amplify the target region with primers to detect the presence of deletions (PARP1-Del-F 5′-AGTGTGCCCTGCGTATTTGC-3′ and PARP1-Del-R 5′-CACAGGGATGAATCTTTCTGGTC-3′) and wild-type alleles (PARP1-In-F 5′-CGCTCCCTTGGTACCACATATG-3′ and PARP1-In-R 5′-GGCTTACTGACAGTCAGCGAAG-3′). Both reactions were run on an S1000 Thermal Cycler (Bio-Rad, Singapore) using BioMaster HS-Taq PCR-Color (2×) (Biolabmix, Novosibirsk, Russia) with the following program: 95 °C for 3 min; 35 cycles: 95 °C for 30 s; 60 °C for 30 s; 72 °C for 30 s; and 72 °C for 3 min. The products of the reactions were resolved in 1% agarose gel stained with ethidium bromide.

## 4. Conclusions

TDP1 promotes the cleavage of the stable DNA–TOP1 complexes with the clinically used anticancer drug topotecan (Tpc), which is a TOP1 inhibitor. TDP1 activity may be a possible cause of tumor resistance to TOP1 inhibitors. A series of new UA thioether and sulfoxide derivatives were synthesized.

The usnic acid thioether and sulfoxide derivatives efficiently suppressed TDP1 activity with the IC_50_ values in the 1.4–25.2 μM range. The structure of the heterocyclic substituent affects the TDP1 inhibitory efficiency of these compounds. Derivatives containing a five-membered heterocyclic fragment itself or fused to a benzene ring (**7b**,**c**,**e**–**g** and **10a**) inhibit TDP1 in the low micromolar concentration range (IC_50_ of 1.4–4.4 μM). Compounds containing a six-membered heterocycle (**7i**–**k**) inhibit TDP1 at higher concentrations (IC_50_ > 11 μM). The presence of a halogen in the *para*-position of the benzene substituent enhances the inhibitory properties of the compounds. For the most effective inhibitors of TDP1 **7g**,**h** and their sulfoxide analogs **10a**,**b**, we observed the uncompetitive type of inhibition. The uncompetitive inhibitors prevent the second step of the reaction stabilizing the enzyme–DNA covalent complex. Thus, uncompetitive TDP1 inhibitors could lead to the accumulation of the single-strand breaks in cancer cells.

The anticancer effect of the TOP1 inhibitors can be significantly enhanced by the simultaneous inhibition of PARP1, TDP1, and TDP2. We tested the ability of the synthesized compounds to inhibit the TDP1, TDP2, and PARP1 activities. We found the compounds act as dual or triple inhibitors of TDP1, TDP2, and PARP1. Some of the compounds inhibited not only TDP1 but also TDP2 and PARP1, but at significantly higher concentration ranges than TDP1.

Interestingly, the sulfoxide analogs **10a**,**b** were less cytotoxic than their thioester analogs **7g**,**h**. Compound **10b** was one of the less toxic among the ten tested compounds. One of the most effective TDP1 inhibitors, **10a** (IC_50_ 2.1 μM, Table 1), also moderately inhibit TDP2, and showed promising synergy on HeLa cells in conjunction with topotecan. That is of great importance for further development of sensitizers to topotecan and other clinically used TOP1 inhibitors.

## Figures and Tables

**Table 1:** TDP1, TDP2, and PARP1 inhibition ability and cytotoxicity of the thioether UA derivatives.

		R	IC_50_ (TDP1), µM	HEK293A CC_50_, µM	HEK293FT CC_50_, µM	HeLa CC_50_, µM	PARP1, 1 mM	TDP2, 1 mM
1	UA	-	>50	ND	ND	20 ± 10	-	-
2	**9**	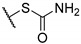	6.6 ± 1.0	>50	7 ± 2	13 ± 1	+	+
3	**7a**	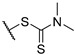	5.4 ± 2.9	>50	>50	13.0 ± 0.5	+	+
4	**7b**	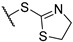	4.4 ± 1.0	>50	16 ± 5	11.0 ± 0.5	+	+
5	**7c**	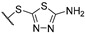	4.3 ± 0.5	>50	35 ± 2	33 ± 2	+	+
6	**7d**	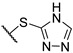	25.2 ± 6.5	ND	ND	ND	+	+
7	**7e**	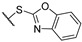	3.2 ± 0.5	26 ± 2	4.5 ± 1.0	2.9 ± 0.2	-	+
8	**7f**	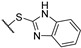	2.4 ± 1.0	>50	10 ± 2	13 ±	-	+
9	**7g**	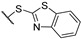	1.7 ± 0.6	3.5 ± 0.3	3 ± 1	2.0 ± 0.6	-	+
10	**10a**	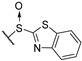	2.1 ± 0.2	20 ± 2	15 ± 2	15 ± 1	-	+
11	**7h**	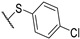	2.2 ± 0.5	10 ± 2	4 ± 1	2.5 ± 0.5	+	+
12	**10b**	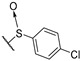	1.4 + 0.2	>50	20 ± 3	27 ± 2	-	+
13	**7i**	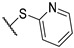	11.9 ± 0.4	ND	ND	ND	-	+
14	**7j**	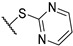	16.9 ± 2.4	ND	ND	ND	+	+
15	**7k**	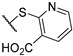	19.6 ± 2.8	ND	ND	ND	+	+

## Supplementary Materials

The following are available online at https://www.mdpi.com/article/10.3390/ijms222111336/s1.
