# Supplementary material for: New Hybrid Compounds Combining Fragments of Usnic Acid and Thioether Are Inhibitors of Human Enzymes TDP1, TDP2 and PARP1
Source: Int J Mol Sci. 2021 Oct 20;22(21):11336. doi: 10.3390/ijms222111336 (PMC8583042; doi:10.3390/ijms222111336)
Supplement: Supplementary file 1 [file ijms-22-11336-s001.zip › ijms-1424134-supplementary.pdf]

Supplementary Material

New hybrid compounds combining fragments of usnic acid and thioether are inhibitors of human enzymes TDP1, TDP2 and PARP1

Nadezhda S. Dyrkheeva 1, Aleksandr S. Filimonov 2, Olga A. Luzina 2, Kristina A. Orlova 3, Irina A. Chernyshova 1, Tatyana E. Kornienko 1, Sergey P. Medvedev 1,4, Alexandra L. Zakharenko 1,5, Anastasia A. Malakhova 1,4, Ekaterina S. Ilina 1, Rashid O. Anarbaev 1, Konstantin N. Naumenko 1, Kristina V. Klabenklova 3,4, Ekaterina A. Burakova 3,4, Dmitry A. Stetsenko 3,4, Suren M. Zakian 1,4, Nariman F. Salakhutdinov 2 and Olga I. Lavrik 1,3,5,*

1 Institute of Chemical Biology and Fundamental Medicine, Siberian Branch of the Russian Academy of Sciences, 630090 Novosibirsk, Russia; dyrkheeva.n.s@gmail.com (N.S.D.); a.zakharenko73@gmail.com (A.L.Z.); katya.plekhanova@gmail.com (E.S.I.); chernyshova0305@gmail.com (I.A.C.); t.kornienko1995@gmail.com (T.E.K.); anarbaev@niboch.nsc.ru (R.O.A.); k-naumenko@mail.ru (K.N.N.)

2 N.N. Vorozhtsov Novosibirsk Institute of Organic Chemistry, Siberian Branch of the Russian Academy of Sciences, 630090 Novosibirsk, Russia; alfil@nioch.nsc.ru (A.S.F.); luzina@nioch.nsc.ru (O.A.L.); anvar@nioch.nsc.ru (N.F.S.)

3 Novosibirsk State University, 630090 Novosibirsk, Russia; kristina-orlova1999@mail.ru (K.A.O.); k.klabenkova@g.nsu.ru (K.V.K.); e.burakova1@nsu.ru (E.A.B.); d.stetsenko@nsu.ru (D.A.S.)

4 Federal Research Centre Institute of Cytology and Genetics, Siberian Branch of the Russian Academy of Sciences, 630090 Novosibirsk, Russia; amal@bionet.nsc.ru (A.A.M.); medvedev@bionet.nsc.ru (S.P.M.); zakian@bionet.nsc.ru (S.M.Z.)

5 Altai State University, 656049, Barnaul, Russia

[*] Correspondence: lavrik@niboch.nsc.ru (O.I.L.)

Content

Figures S1-S23. NMR and DFS spectra of the products 3

Figure S24. MALDI-TOF MS spectrum of 5'-Tyr-AAC GTC AGG GTC TTC C-FAM 14

Table S1. Dependence of TDP1 reaction values Vmax and KM on the concentration of inhibitors 7g, 7h, 10a, 10b 15

Figure S25. Dependence of TDP1 reaction kinetic parameters on the concentration of oligonucleotide substrate and of inhibitors 7g, 7h, 10a, 10b. The illustration of uncompetitive type of inhibition by Lineweaver-Burk (left, (1/v;1/[S])) and Dixon plots (right, (1/v; [I])) 15

Figure S26. The compounds 7g, 7h, 10a, 10b inhibit TDP2 16

Figure S27. PCR analysis of HEK293A PARP1-/- cell line clone 1A3 16

Figure S28. Topotecan (Tpc) cytotoxicity in HEK293A WT and PARP1 -/- cells—dose-dependent action of Tpc by colorimetric test 17

Figure S29. Purification of human recombinant tyrosyl-DNA phosphodiesterase 2 (TDP2) 17

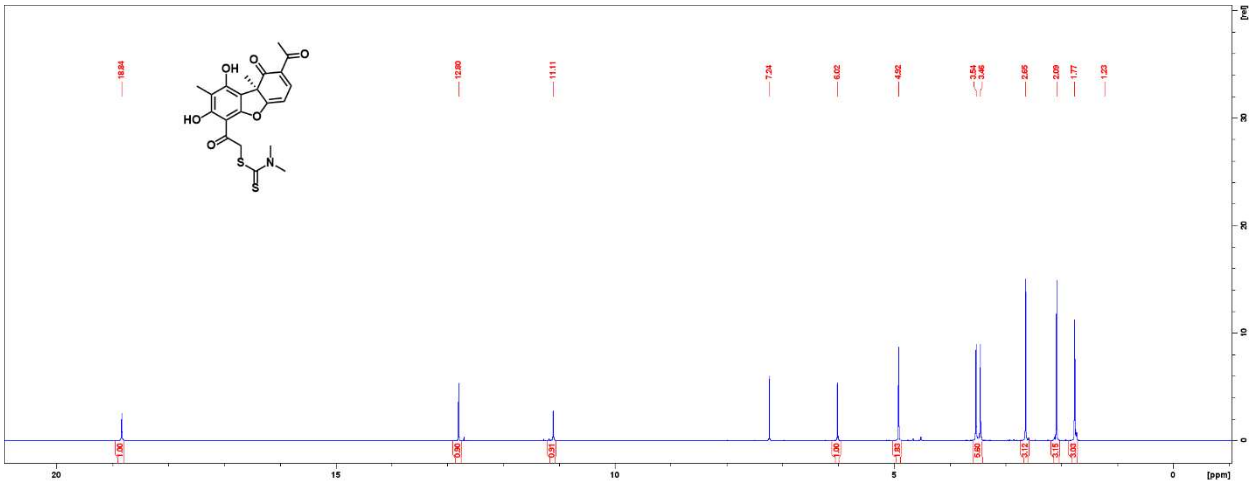

Figure S1 The NMR ^{1}H spectrum of 7a

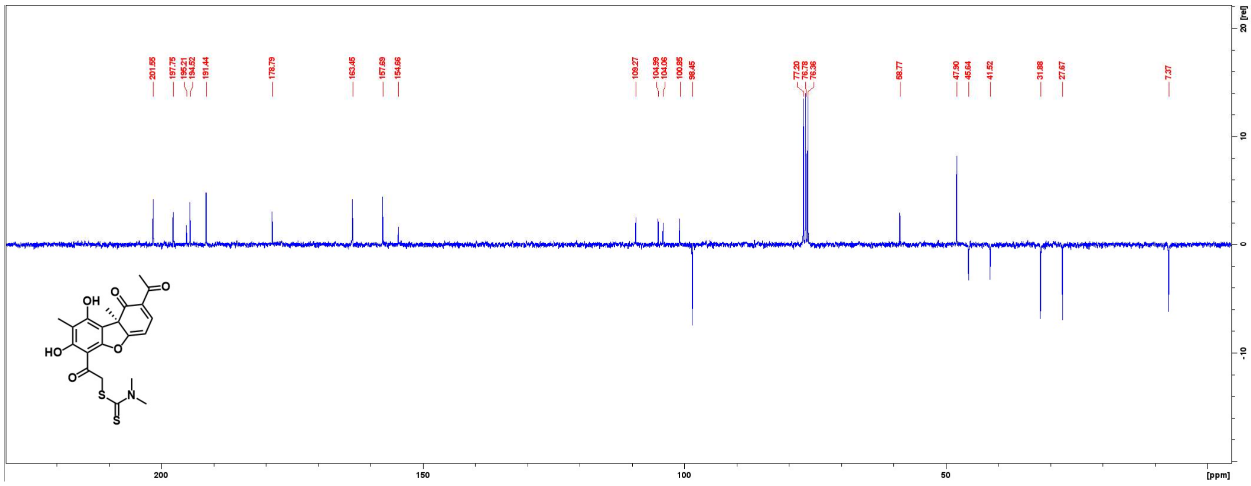

Figure S2 The NMR ^{13}C spectrum of 7a

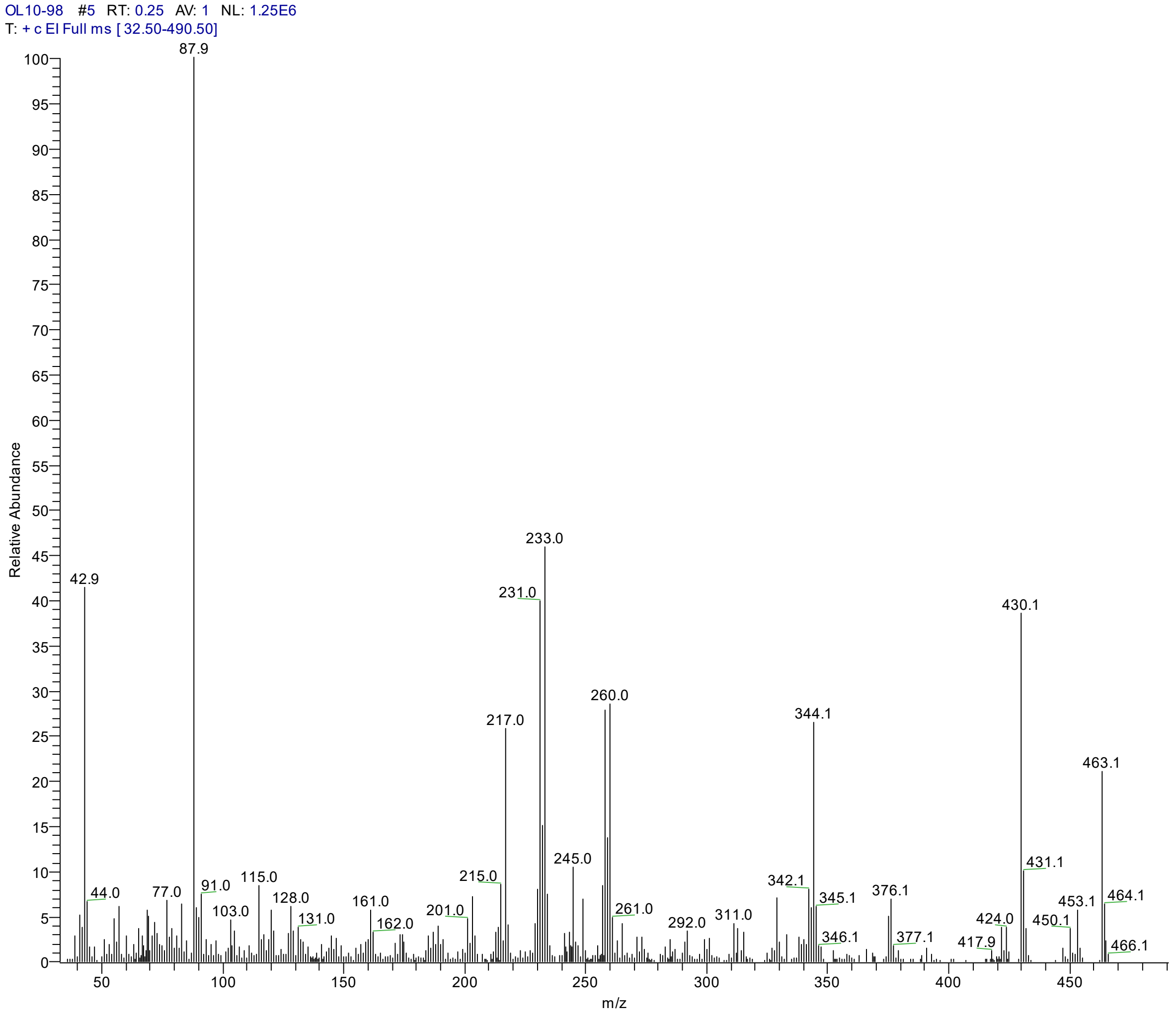

Figure S3 The DFS spectrum of 7a

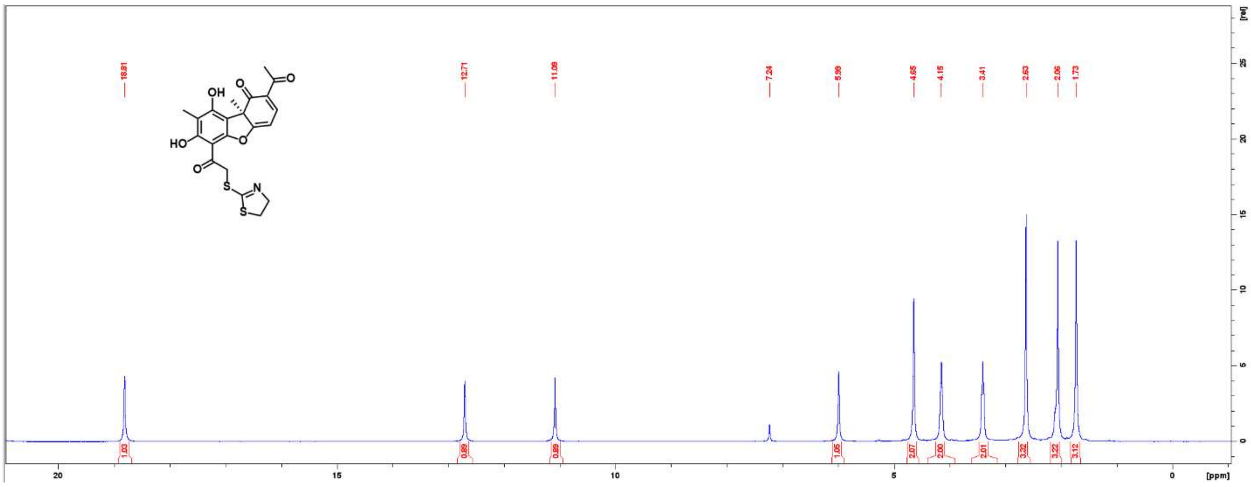

Figure S4 The NMR ^{1}H spectrum of 7b

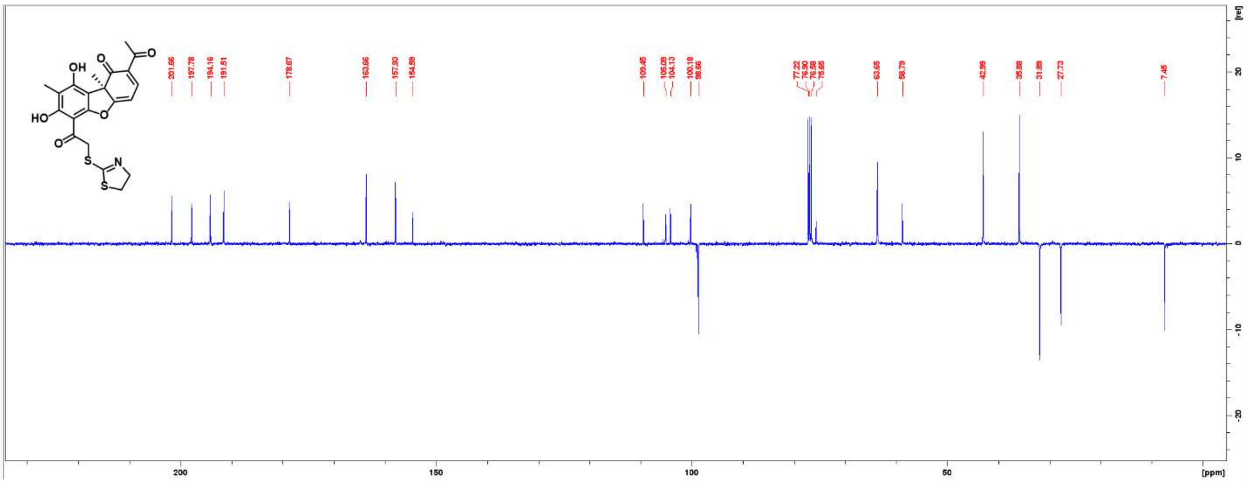

Figure S 5 The NMR ^{13}C spectrum of 7b

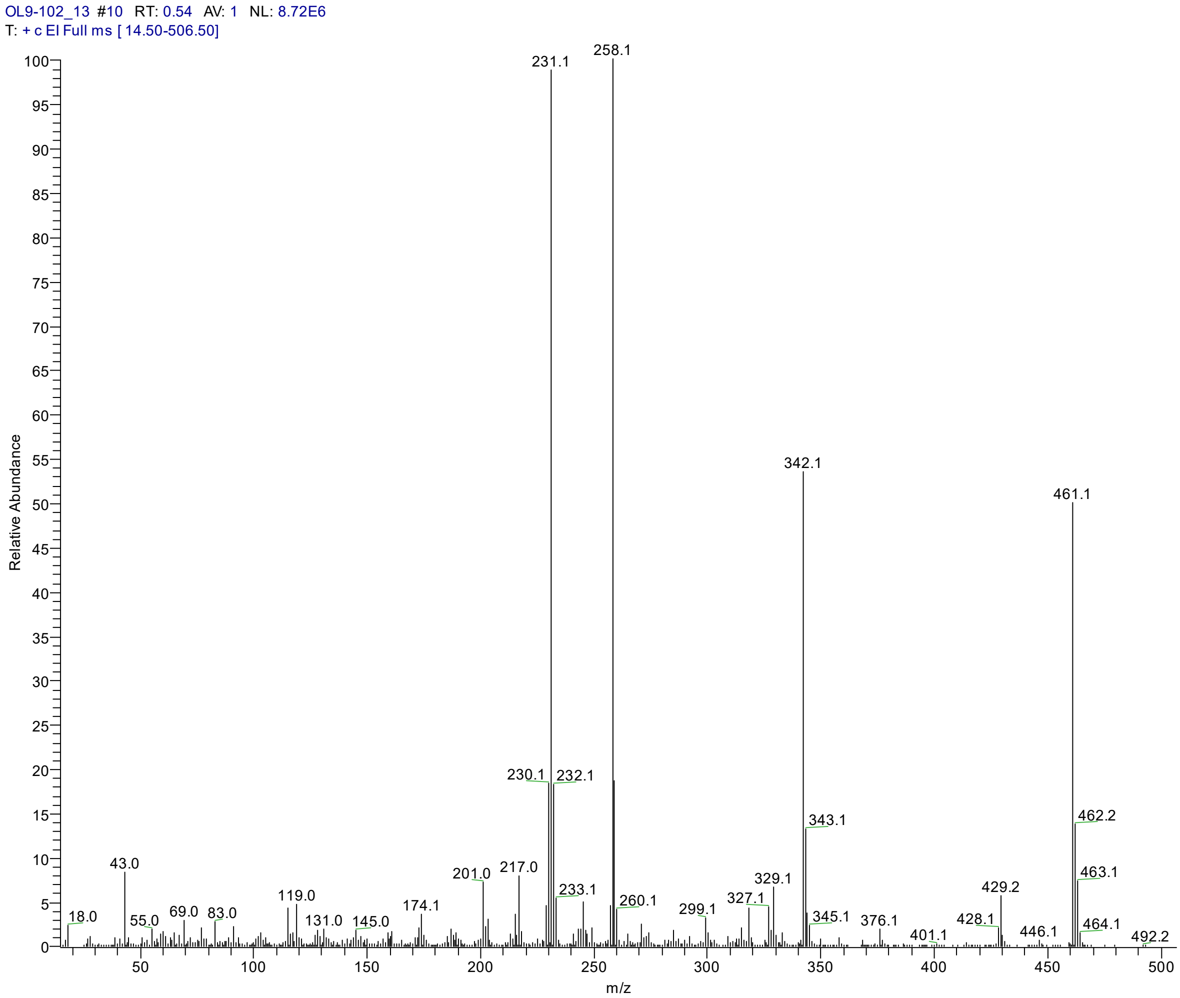

Figure S6 The DFS spectrum of 7b

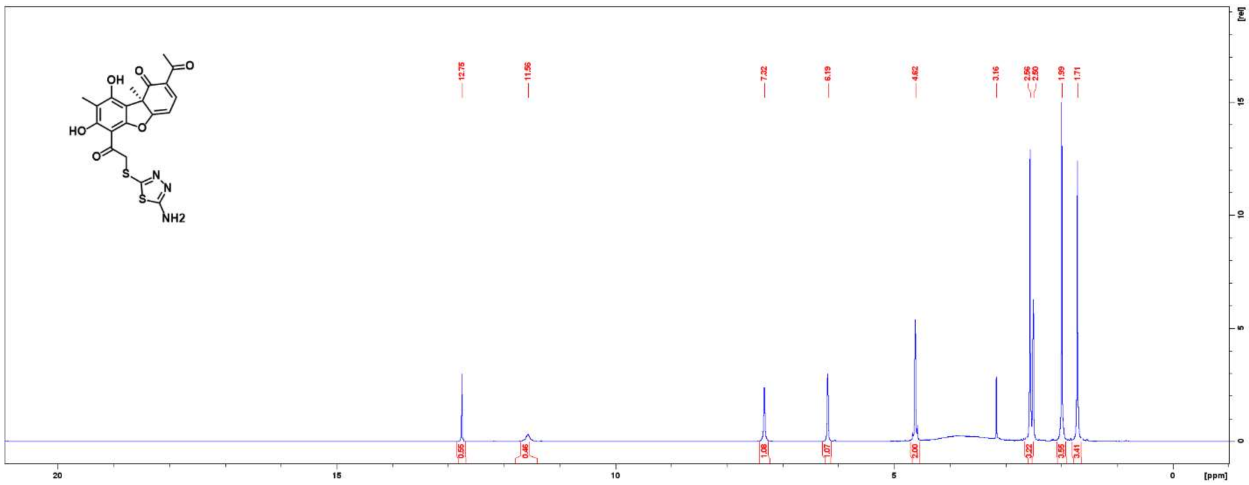

Figure S7 The NMR ^{1}H spectrum of 7c

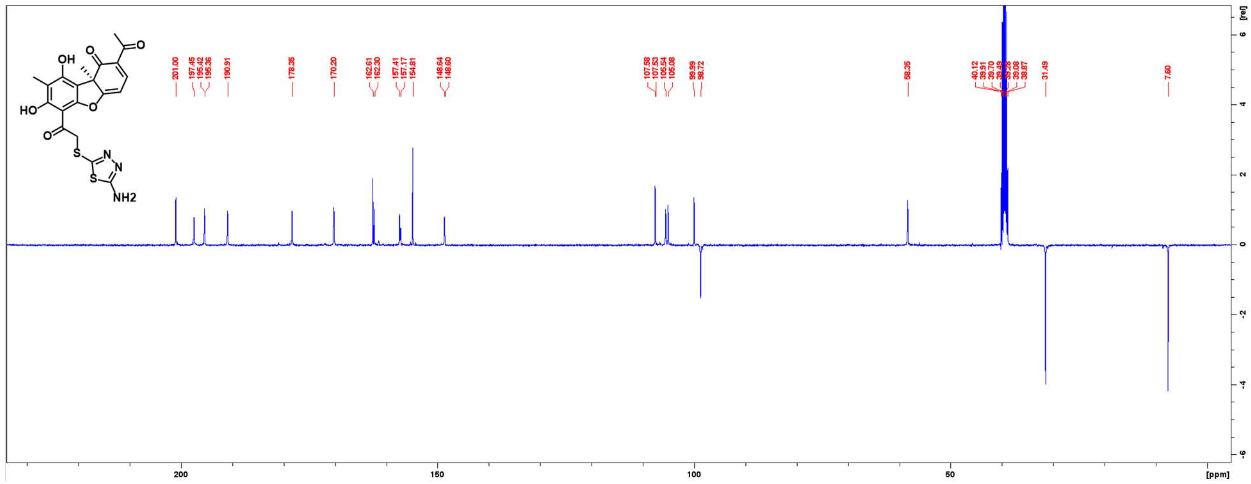

Figure S8 The NMR ^{13}C spectrum of 7c

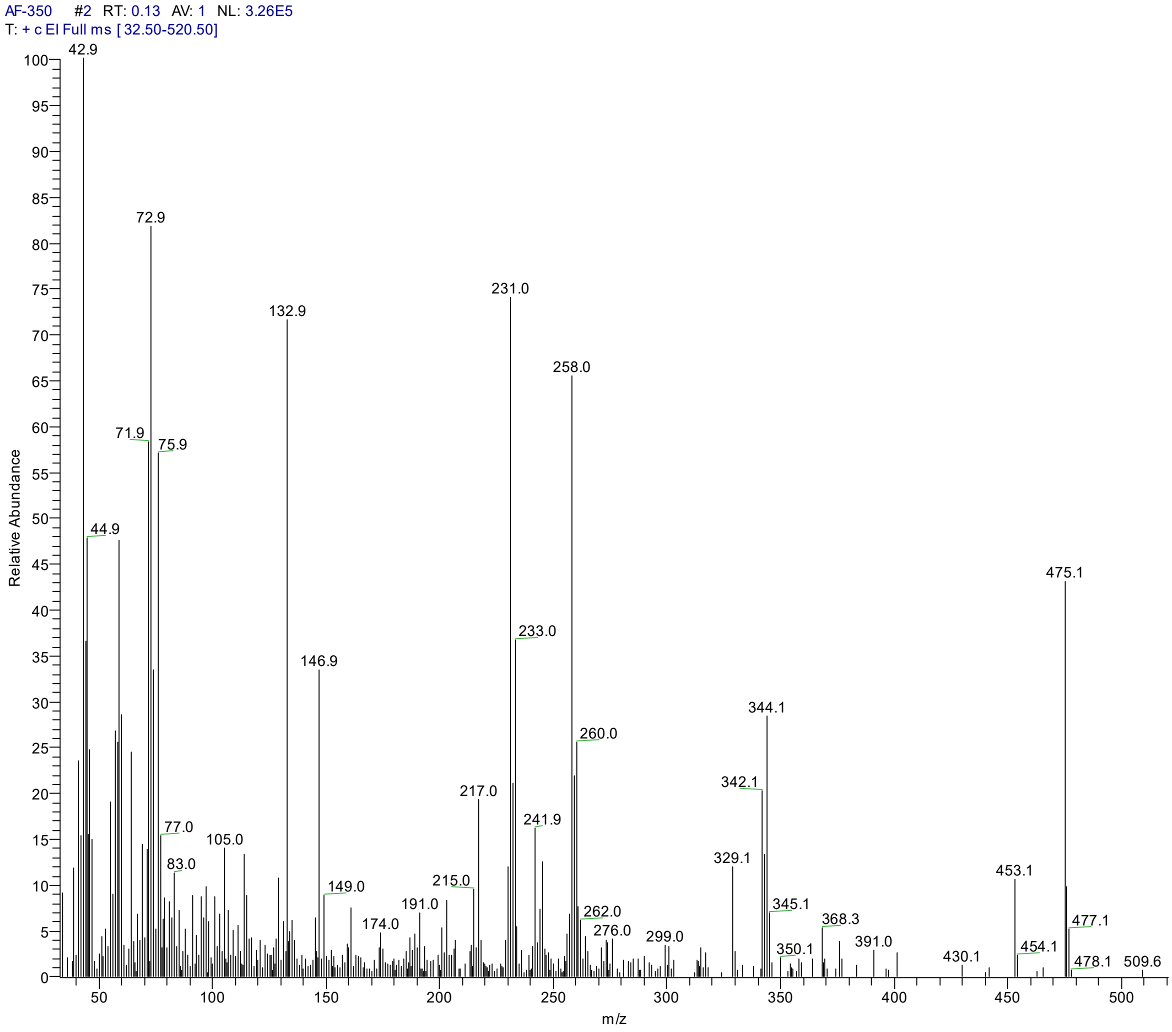

Figure S9 The DFS spectrum of 7c

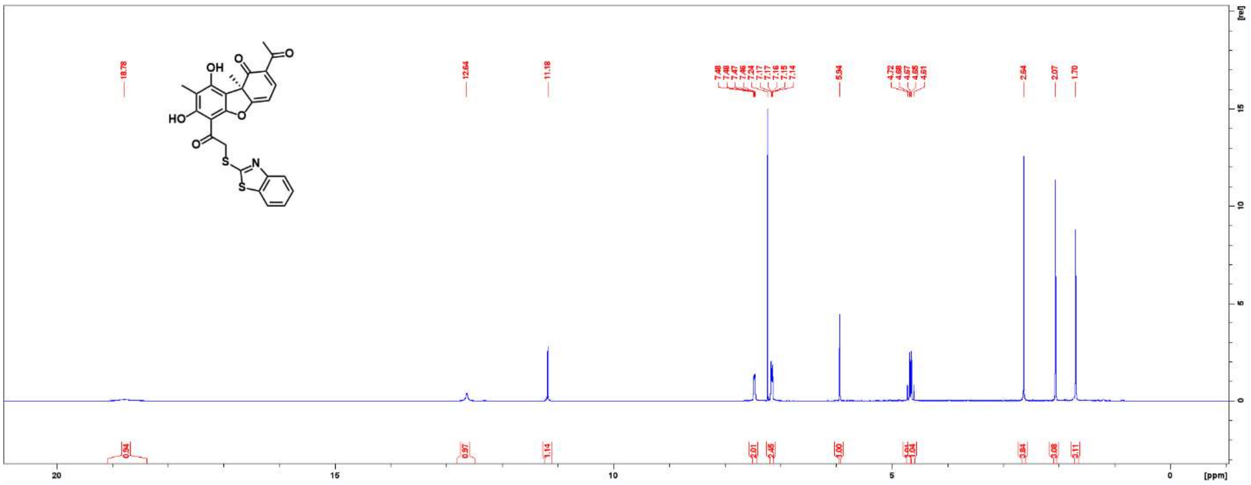

Figure S10 The NMR ^{1}H spectrum of 7f

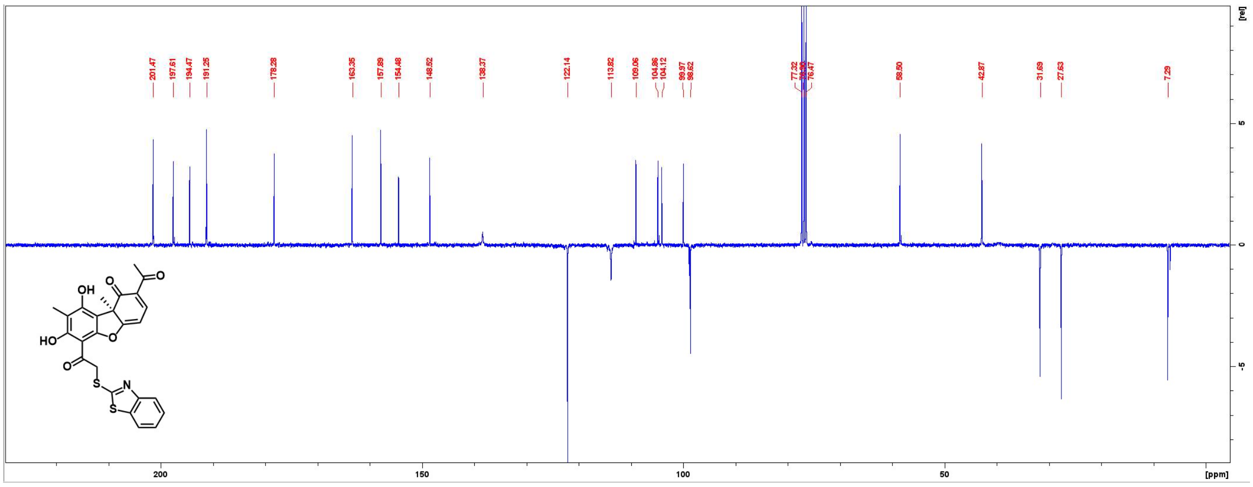

Figure S11 The NMR ^{13}C spectrum of 7f

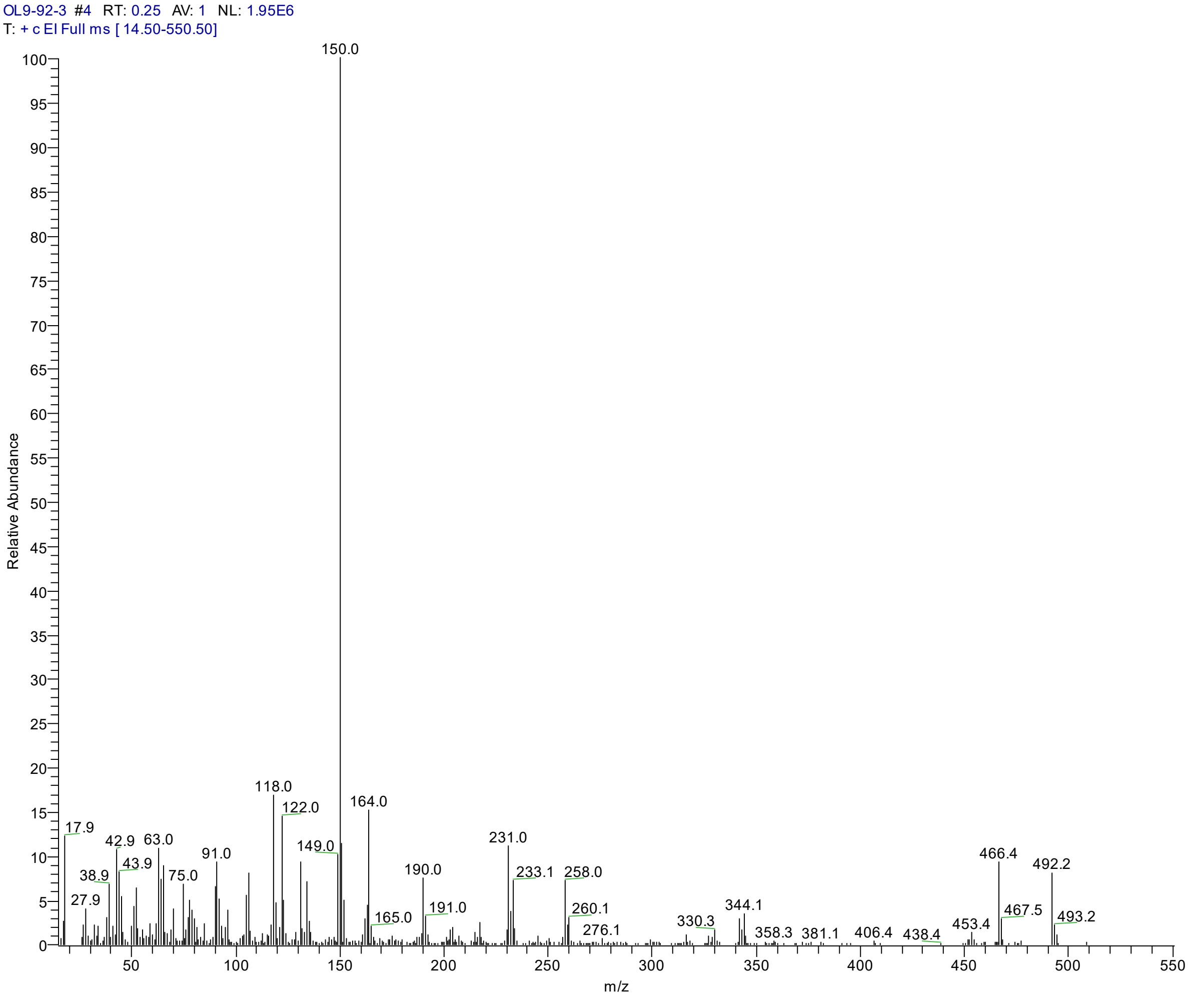

Figure S12 The DFS spectrum of 7f

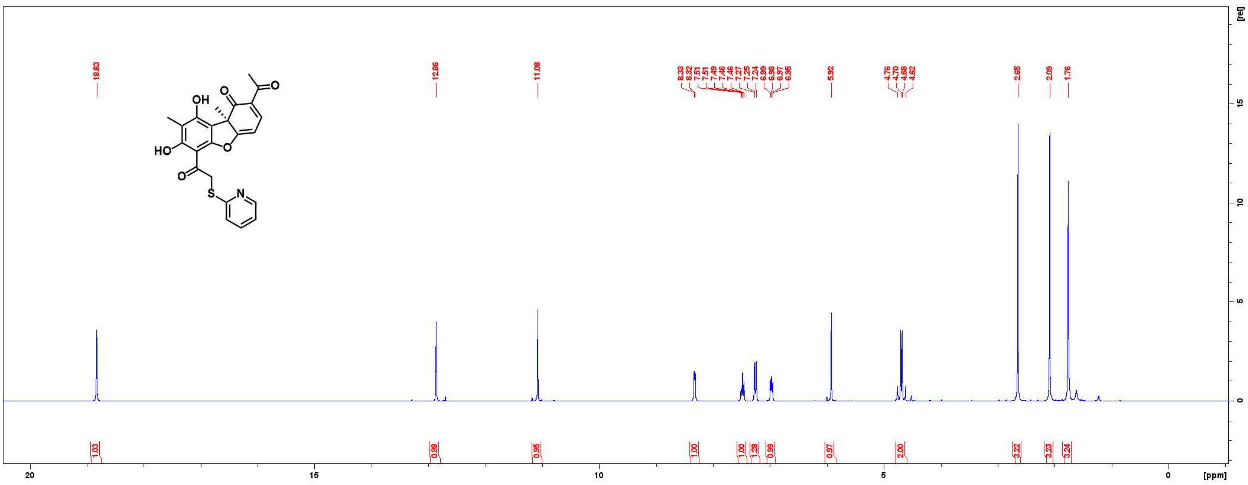

Figure S13 The NMR ^{1}H spectrum of 7i

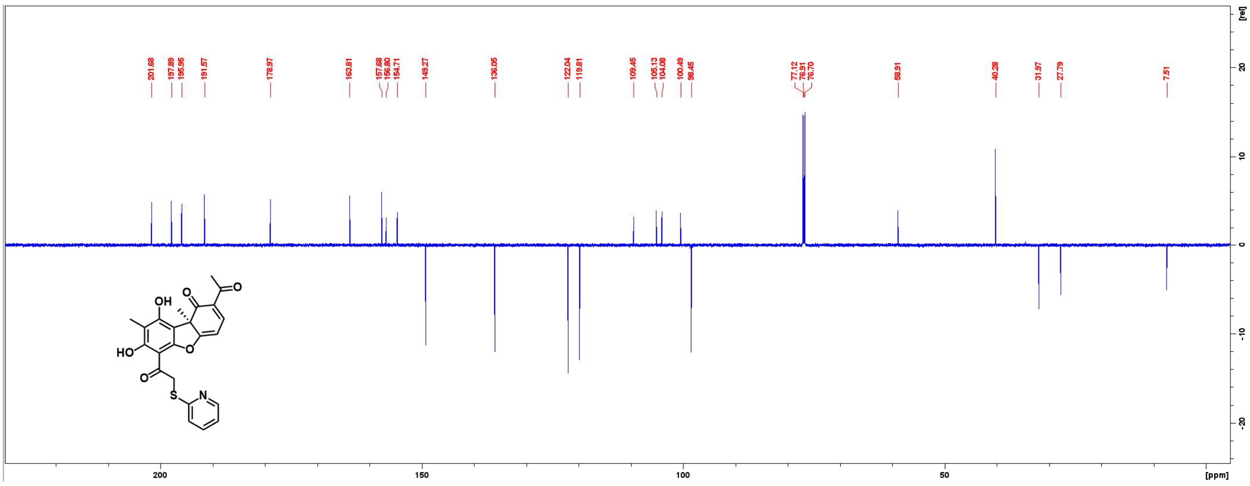

Figure S14 The NMR ^{13}C spectrum of 7i

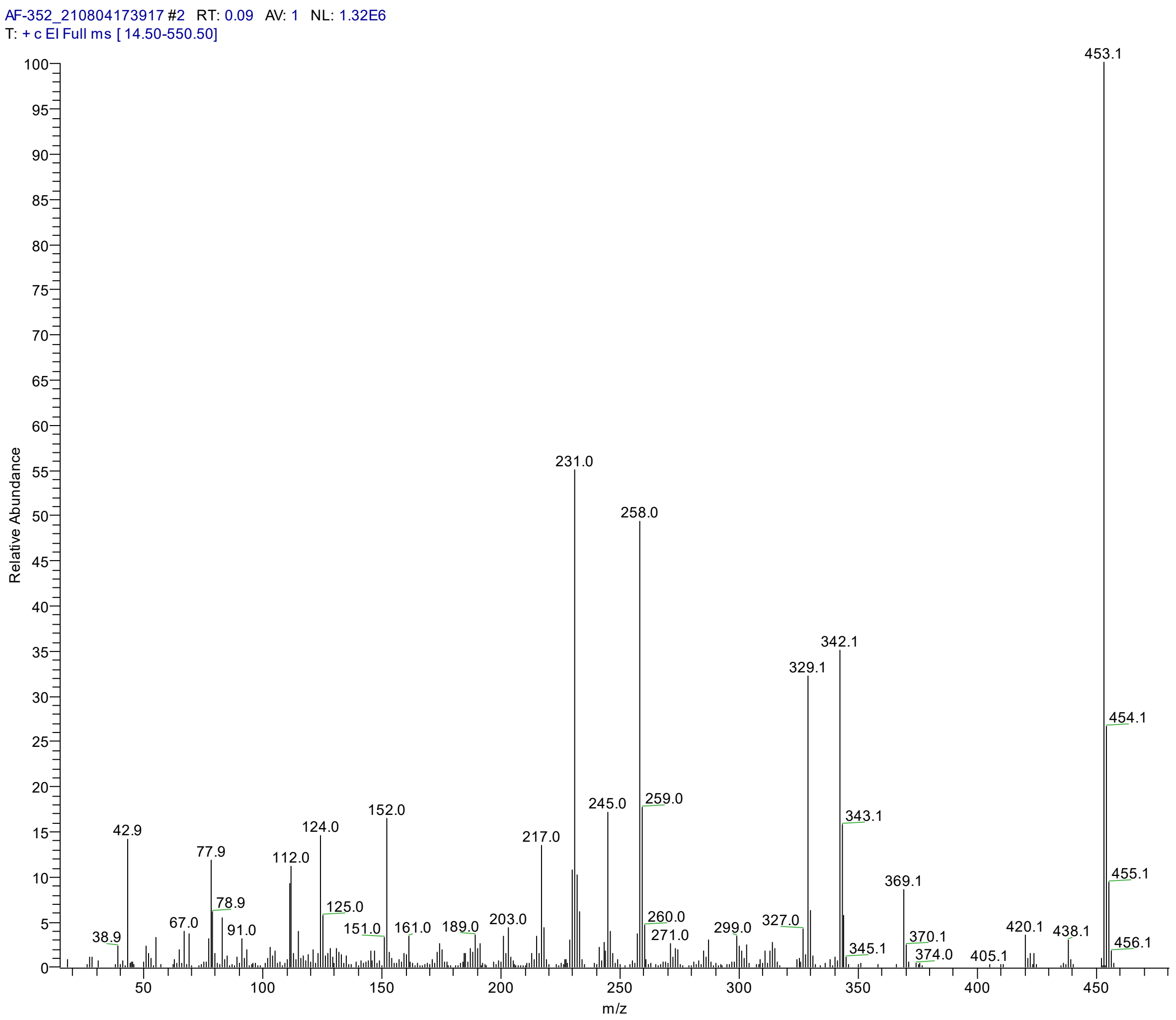

Figure S15 The DFS spectrum of 7i

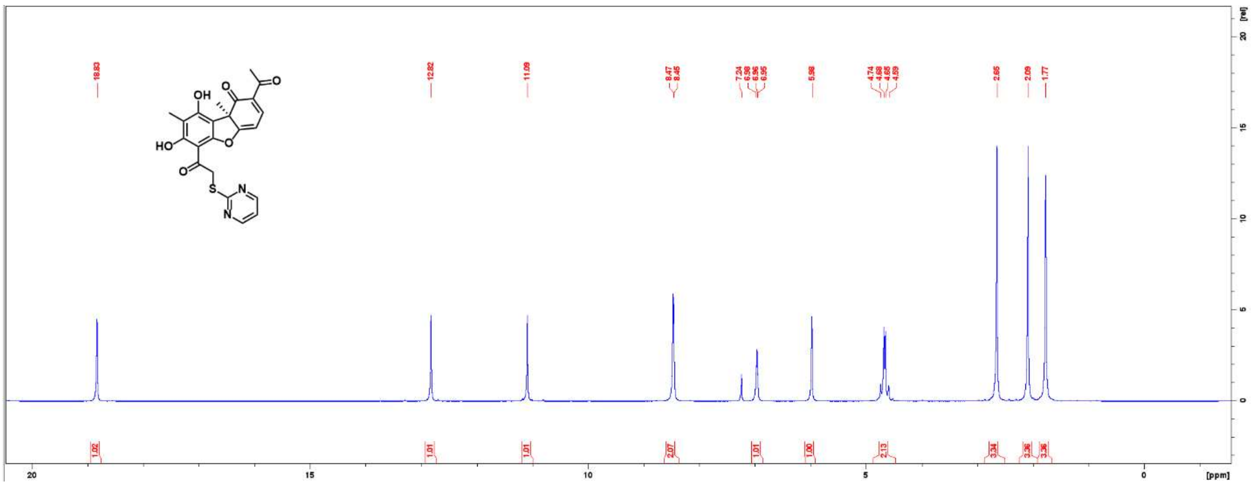

Figure S16 The NMR ^{1}H spectrum of 7j

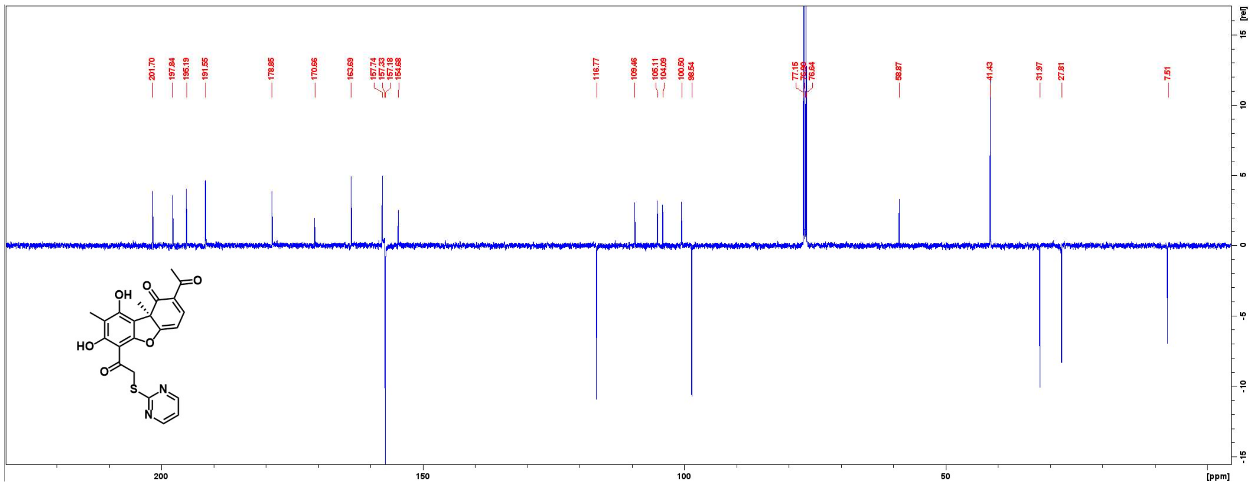

Figure S17 The NMR ^{13}C spectrum of 7j

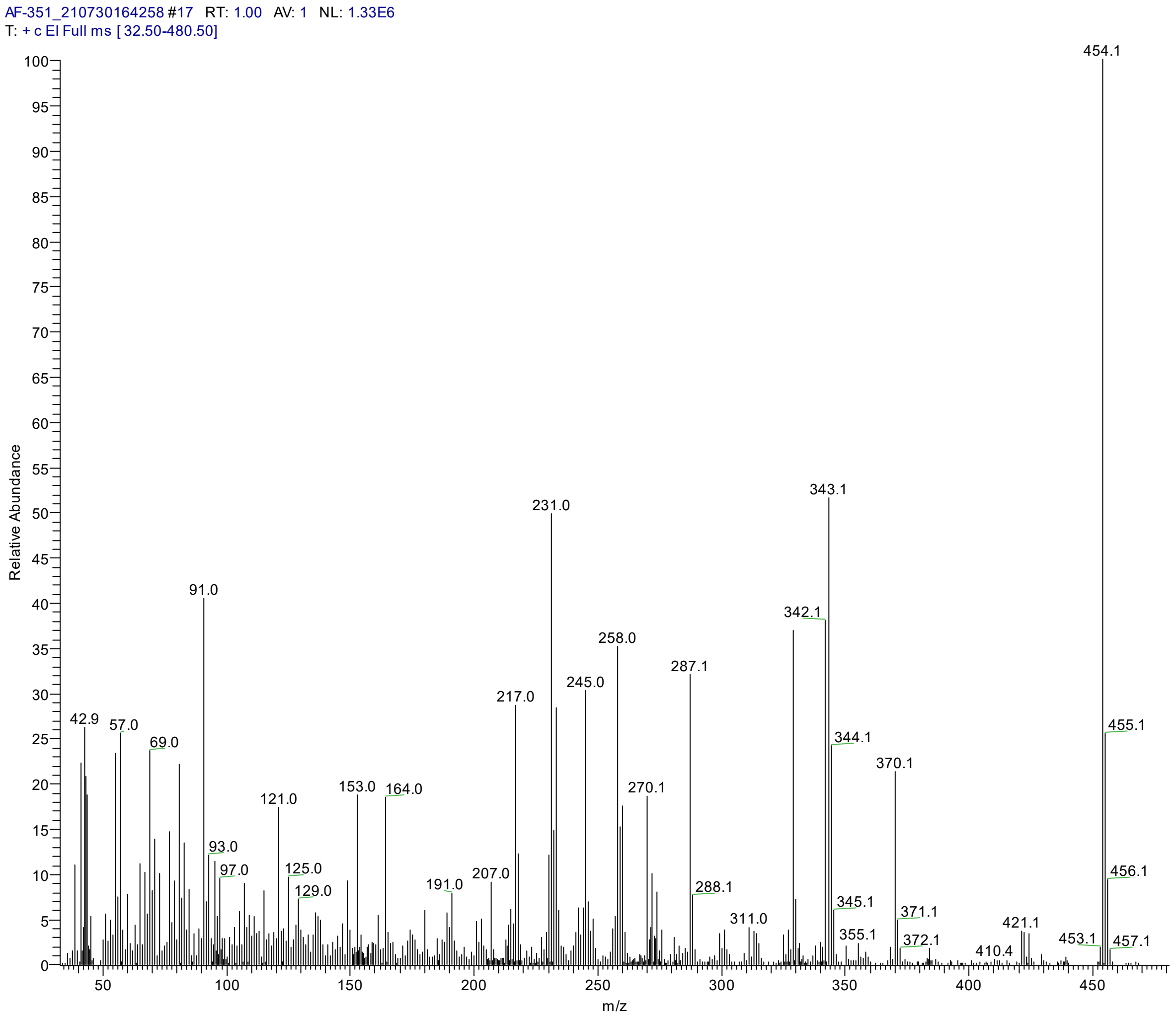

Figure S18 The DFS spectrum of 7j

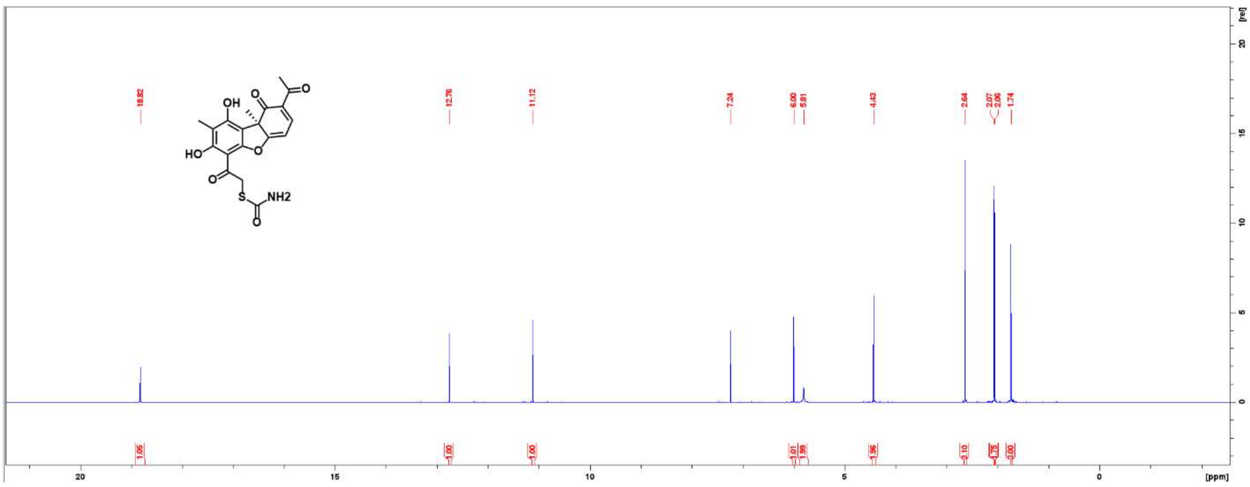

Figure S19 The NMR ^{1}H spectrum of 9

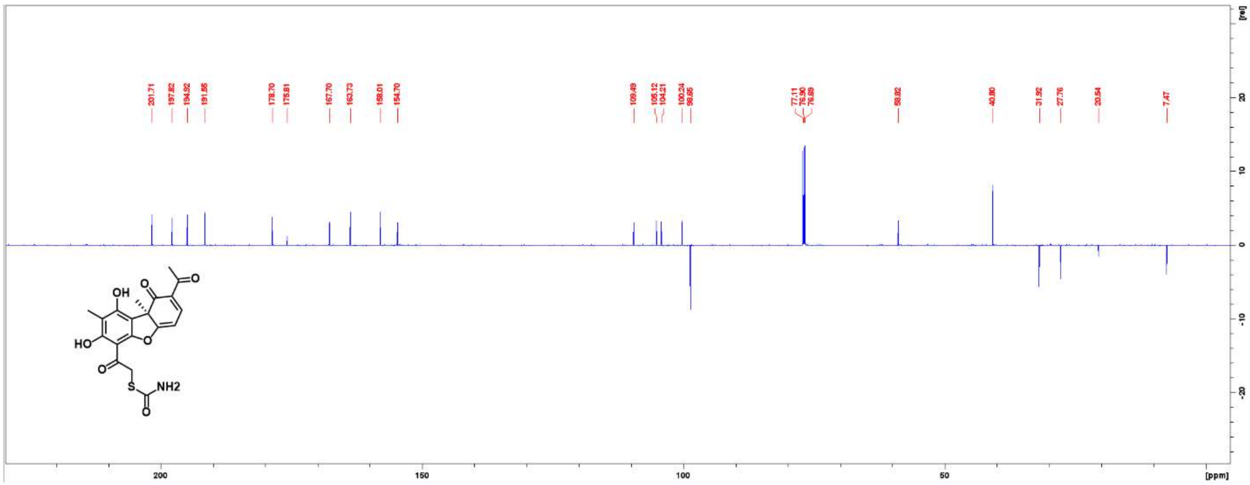

Figure S20 The NMR ^{13}C spectrum of 9

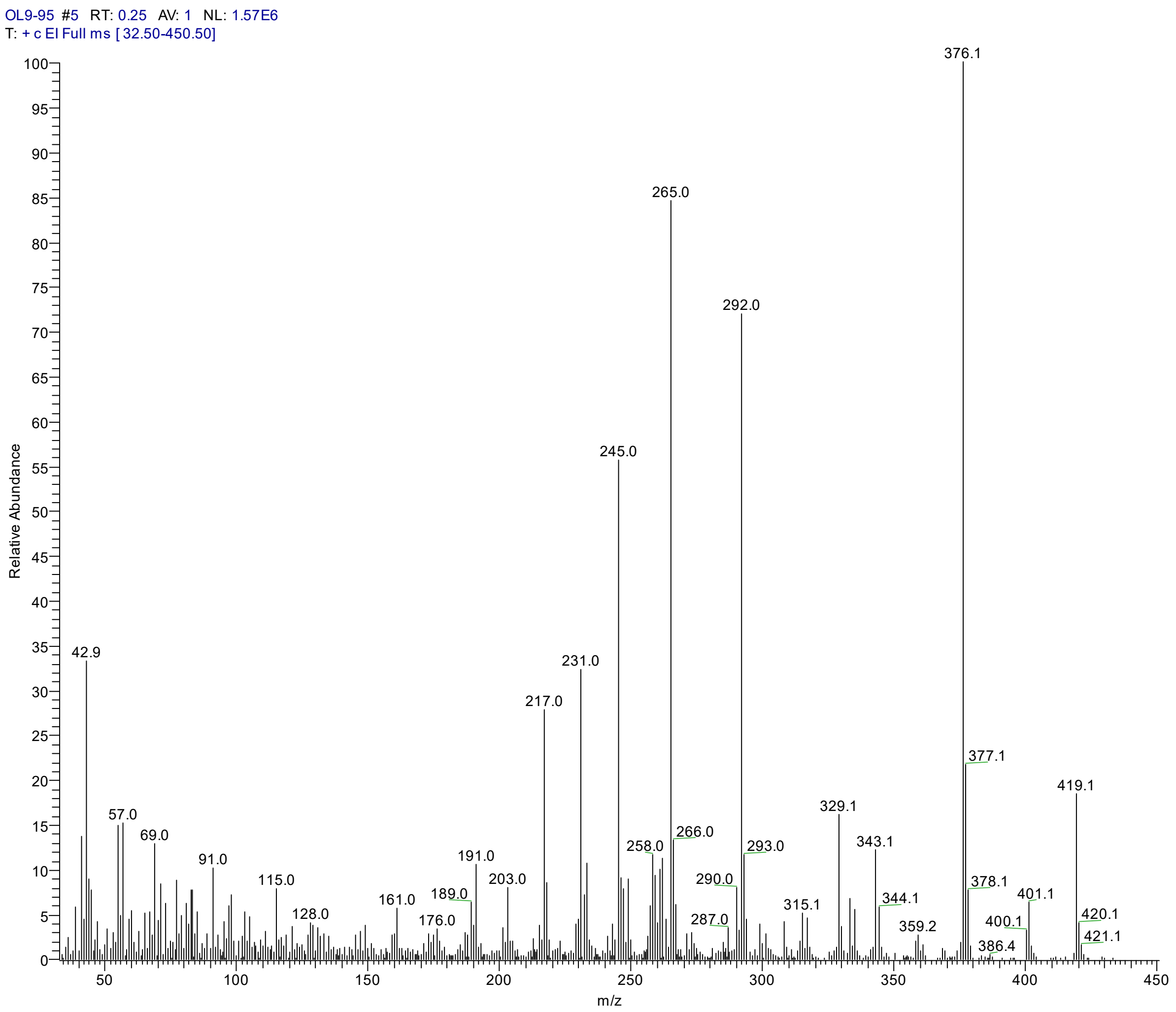

Figure S21 The DFS spectrum of 9

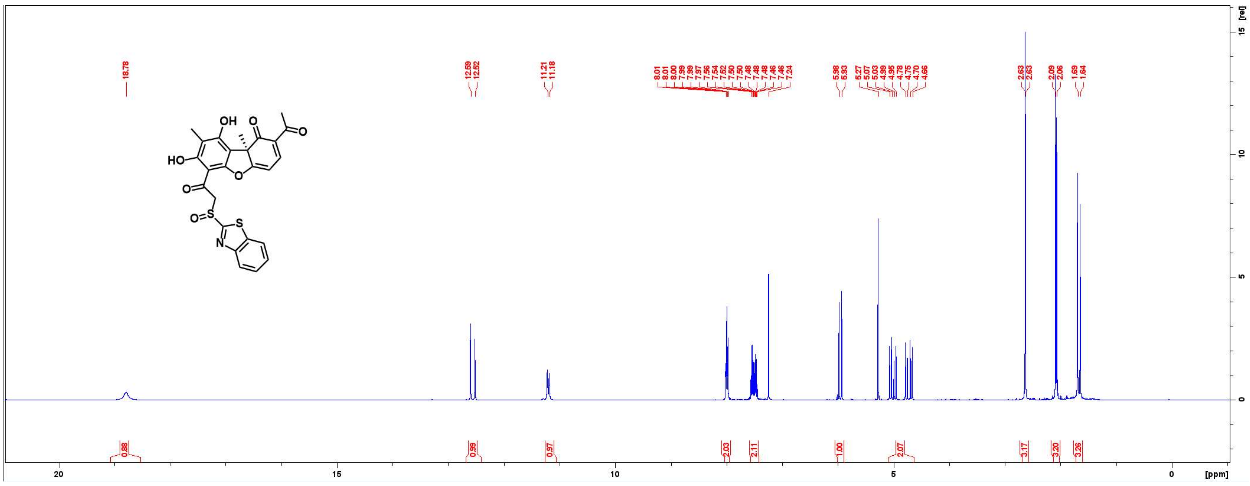

Figure S22 The NMR ^{1}H spectrum of 10a

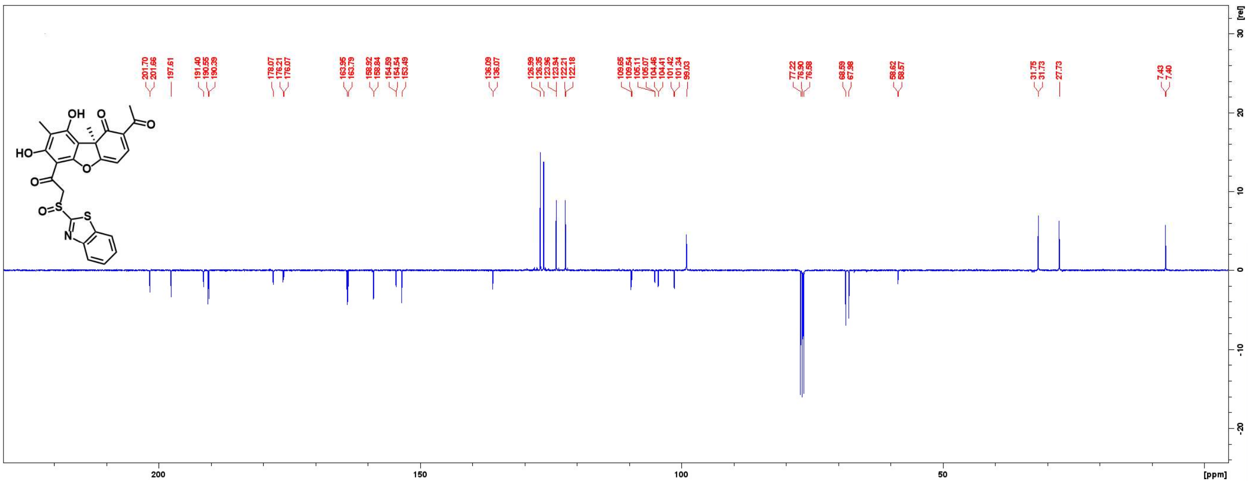

Figure S23 The NMR ^{13}C spectrum of 10a

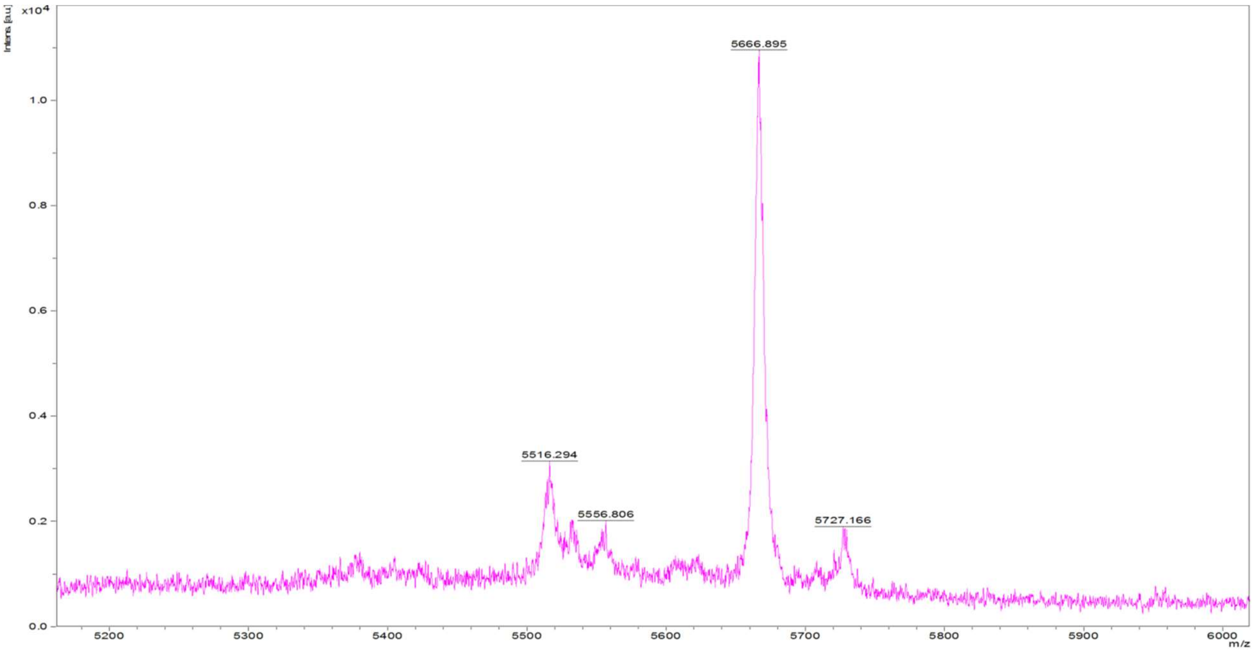

Figure S24 MALDI-TOF MS spectrum of 5'-Tyr-AAC GTC AGG GTC TTC C-FAM.

Table S25 Dependence of TDP1 reaction values V_{max} and K_M on the concentration of inhibitors 7g, 7h, 10a, 10b

[7g], mM	V_{max}	Standard Error	K_M	Standard Error
0	38.8	3.7	156.9	32.7
0.4	35.4	1.3	158.4	10.4
0.8	25.6	2.3	110.7	25.6
1.6	23.0	3.3	134.7	32.9
3.2	19.4	1.1	127.6	13.5
6.4	10.9	0.7	92.1	28.1

[7h], mM	V_{max}	Standard Error	K_M	Standard Error
0	36.3	2.2	205.6	32.8
0.4	31.0	1.9	167.1	29.2
0.8	26.7	1.6	135.2	24.7
1.6	18.5	1.6	93.9	27.9
3.2	15.3	3.3	92.1	30.7
6.4	11.2	1.0	74.8	26.4

[10a], mM	V_{max}	Standard Error	K_M	Standard Error
0	32,1	0.6	174.0	8.6
0.8	27.8	0.5	154.6	7.6
1.6	26.5	0.8	146.5	12.7
3.2	25.1	0.8	144.2	12.4
6.4	21.3	0.4	87.2	5.3

[10b], mM	V_{max}	Standard Error	K_M	Standard Error
0	24.0	2.4	176.8	49.3
0.2	18.7	1.5	127.8	31.1
0.4	10.7	1.4	55.2	31.3
0.8	8.5	1.0	46.1	25.5
1.6	6.3	1.0	21.3	26.1

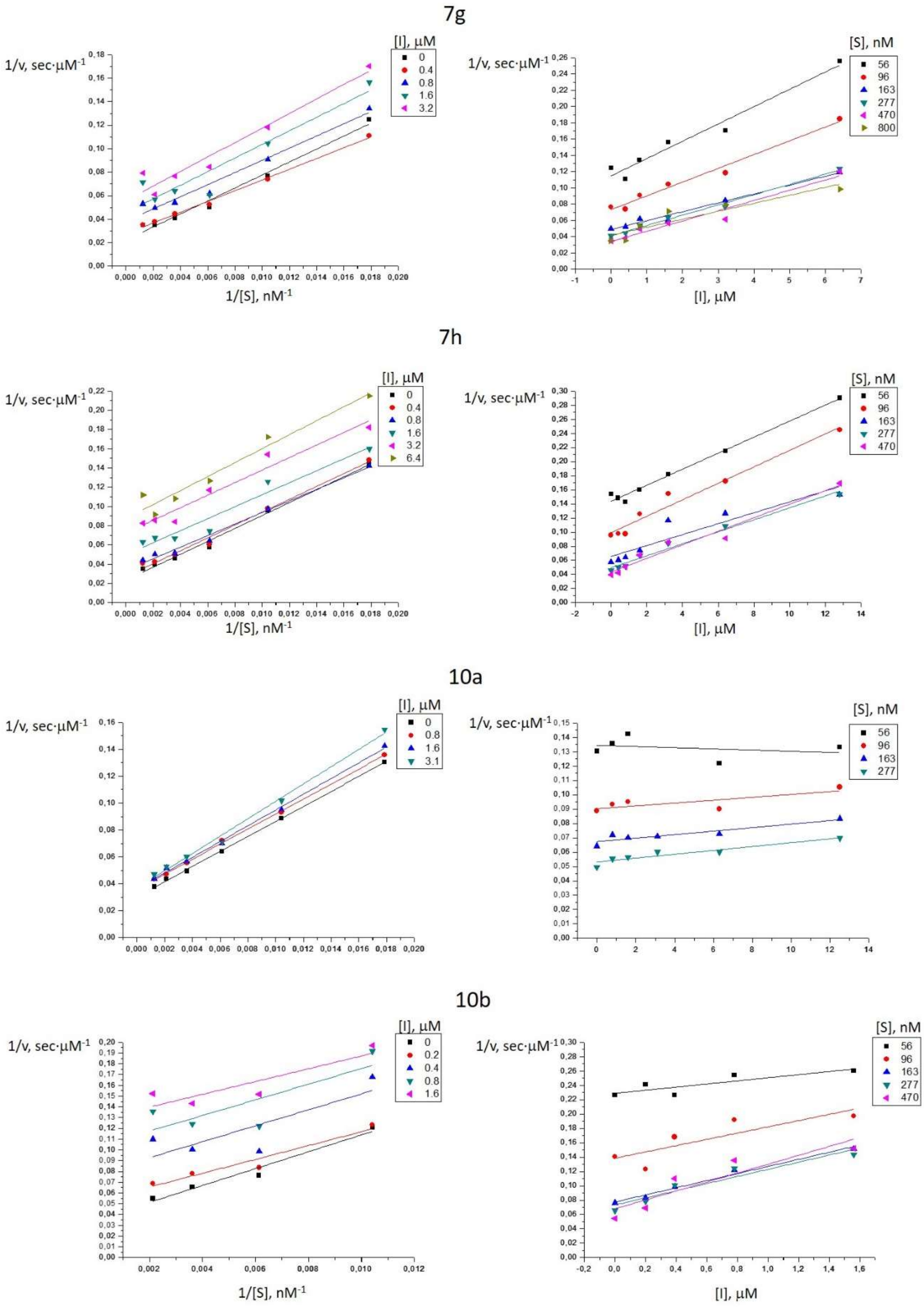

Figure S26 Dependence of TDP1 reaction kinetic parameters on the concentration of oligonucleotide substrate and of inhibitors 7g, 7h, 10a, 10b. The illustration of uncompetitive type of inhibition by Lineweaver-Burk (left, (1/v;1/[S])) and Dixon plots (right, (1/v; [I])).

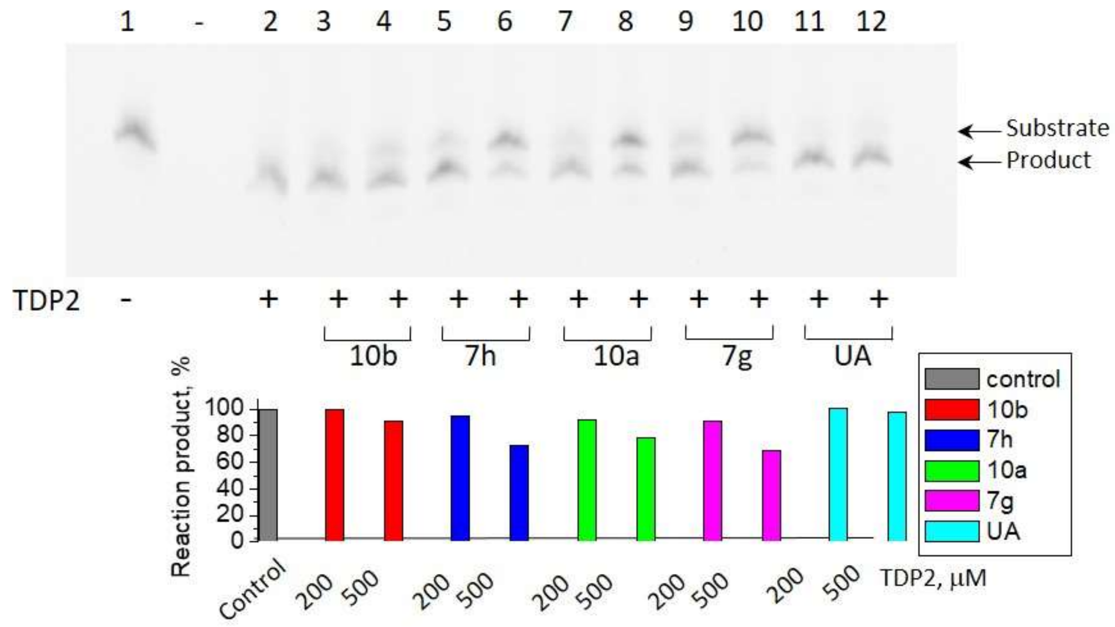

Figure S27 The compounds 7g, 7h, 10a, 10b inhibit TDP2.

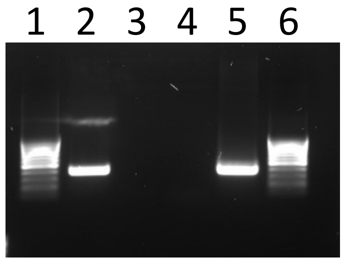

Figure S28 PCR analysis of HEK293A PARP1-/- cell line clone 1A3. 1) 100 bp DNA leader; 2) PCR with HEK293A clone 1A3 gDNA and the primers for detection of deletion in the PARP1 gene; 3) PCR with HEK293A clone 1A3 gDNA and the primers for detection of the PARP1 wild-type allele; 4) PCR with intact HEK293A gDNA and the primers for detection of deletion in the PARP1 gene; 5) PCR with intact HEK293A gDNA and the primers for detection of the PARP1 wild-type allele; 6) 100 bp DNA leader.

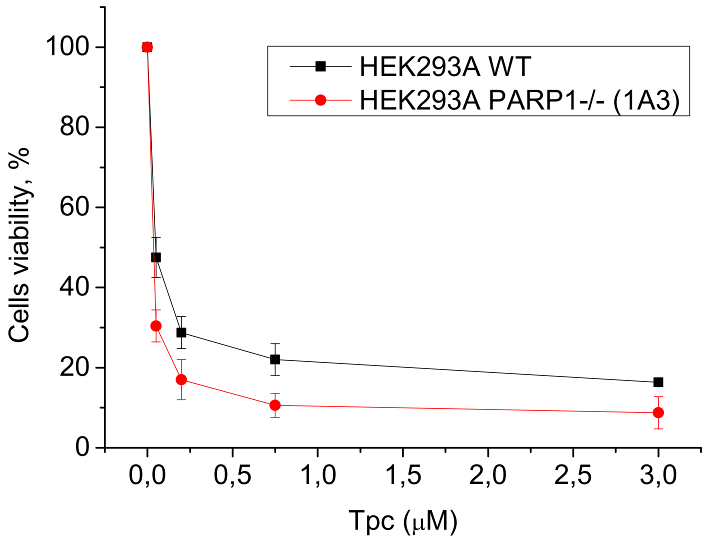

Figure S28 Topotecan (Tpc) cytotoxicity in HEK293A WT and PARP1 -/- cells—dose-dependent action of Tpc by colorimetric test.

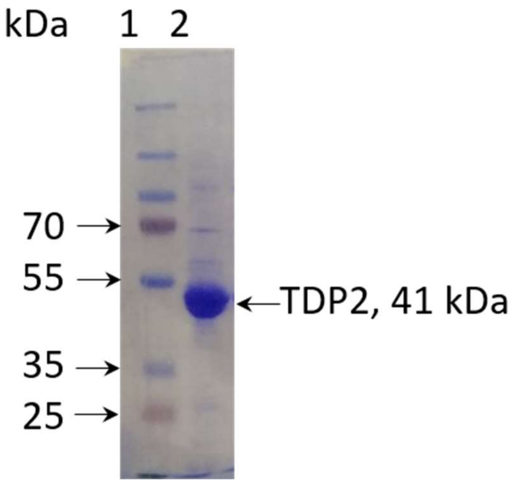

Figure S29 Purification of human recombinant tyrosyl-DNA phosphodiesterase 2 (TDP2).
